# Splice variants of CK1α and CK1α-like: Comparative analysis of subcellular localization, kinase activity, and function in the Wnt signaling pathway

**DOI:** 10.1016/j.jbc.2024.107407

**Published:** 2024-05-23

**Authors:** Tomáš Gybeľ, Štěpán Čada, Darja Klementová, Martin P. Schwalm, Benedict-Tilman Berger, Marek Šebesta, Stefan Knapp, Vítězslav Bryja

**Affiliations:** 1 Department of Experimental Biology, Faculty of Science, Masaryk University, Brno, Czech Republic; 2 CEITEC-Central European Institute of Technology, Masaryk University, Brno, Czech Republic; 3 Institute for Pharmaceutical Chemistry, Johann Wolfgang Goethe-University, Frankfurt am Main, Germany; 4 Structural Genomics Consortium, Johann Wolfgang Goethe-University, Frankfurt am Main, Germany; 5 German Cancer Consortium (DKTK)/German Cancer Research Center (DKFZ), DKTK Site Frankfurt-Mainz, Heidelberg, Germany; 6 Department of Cytokinetics, Institute of Biophysics of the Czech Academy of Sciences, Brno, Czech Republic

**Keywords:** casein kinase 1 alpha (CK1α), casein kinase 1 alpha-like (CK1α-like), Wnt pathway, β-catenin, Axin, phosphorylation, alternative splicing, gene knockout, NanoBRET, inhibitor

## Abstract

Members of the casein kinase 1 (CK1) family are important regulators of multiple signaling pathways. CK1α is a well-known negative regulator of the Wnt/β-catenin pathway, which promotes the degradation of β-catenin *via* its phosphorylation of Ser45. In contrast, the closest paralog of CK1α, CK1α-like, is a poorly characterized kinase of unknown function. In this study, we show that the deletion of CK1α, but not CK1α-like, resulted in a strong activation of the Wnt/β-catenin pathway. Wnt-3a treatment further enhanced the activation, which suggests there are at least two modes, a CK1α-dependent and Wnt-dependent, of β-catenin regulation. Rescue experiments showed that only two out of ten naturally occurring splice CK1α/α-like variants were able to rescue the augmented Wnt/β-catenin signaling caused by CK1α deficiency in cells. Importantly, the ability to phosphorylate β-catenin on Ser45 in the *in vitro* kinase assay was required but not sufficient for such rescue. Our compound CK1α and GSK3α/β KO models suggest that the additional nonredundant function of CK1α in the Wnt pathway beyond Ser45-β-catenin phosphorylation includes Axin phosphorylation. Finally, we established NanoBRET assays for the three most common CK1α splice variants as well as CK1α-like. Target engagement data revealed comparable potency of known CK1α inhibitors for all CK1α variants but not for CK1α-like. In summary, our work brings important novel insights into the biology of CK1α, including evidence for the lack of redundancy with other CK1 kinases in the negative regulation of the Wnt/β-catenin pathway at the level of β-catenin and Axin.

Protein kinases, studied since the mid-1950s, have emerged now as the most critical and widely studied cellular signaling molecules (1). The human kinome was initially described as a group of 518 protein kinases that comprises approximately 2% of human genes (2). Protein kinases constitute one of the largest and most functionally diverse protein families. The family of casein kinase 1 (CK1) was among the first kinases described in the literature (3).

CK1 family members are serine/threonine protein kinases that are highly conserved from yeast to human (4). Individual family members share high level of similarity between paralogs; kinase domains show highest level of homology (51–98%) whereas the largest differences characterizing individual paralogs are in the N- and C-terminal regions. In human, seven CK1 paralogs have been reported, namely CK1alpha (CK1α), CK1alpha-like (CK1α-like), CK1gamma1 (CK1γ1), CK1gamma2 (CK1γ2), CK1gamma3 (CK1γ3), CK1delta (CK1δ), and CK1epsilon (CK1ε). Of note, in some mammals (*Bos taurus*), another CK1 paralog—CK1beta (CK1β)—is present but this gene is missing in human and mouse genomes. Members of the CK1 family are ubiquitously expressed, but their expression levels differ in individual tissues and cell types (5, 6).

CK1α is a multifunctional protein encoded in humans by the *CSNK1A1* gene, located on the chromosome 5q32. The gene has been reported to be expressed as four alternatively-spliced transcript variants that are translated into four protein isoforms (for review, see Jiang *et al.* (7)). The different CK1α isoforms vary in length due to a 12-amino acid “S” insert near the C-terminus and a 28-amino acid “L” insert in the kinase domain. The latter has been described as unique to vertebrate CK1α isoforms (8) and it contains the sequence motif PVGKRKR, which is a nuclear localization signal (NLS). CK1α has been shown to be catalytically active even with the L-segment insertion into the kinase domain (9). Both insertions contain several serine and threonine residues flanked by basic amino acids, suggesting the presence of *in vivo* site-specific phosphorylation (10). The 337 amino acids isoform is the predominant CK1α variant (UniProtKB - P48729) with a known three dimensional structure determined by crystallography (PDB: 5FQD, 6GZD) (11, 12).

Compared to CK1α, its close paralog CK1α-like, encoded by *CSNK1A1L* gene, is poorly characterized. CK1α and CK1α-like share 90.5% homology across the whole protein sequence. Differences are mainly located in the N- and C-terminal regions. Recently CK1α-like was included in the Dark Kinase Knowledgebase (13), a project focusing on understudied kinases.

CK1α is known to be associated with a plethora of cellular processes and represents a potential therapeutic target in cancer (for review, see Janovská *et al.* (14); Jiang *et al.* (7)). One of the best-known functions of CK1α is its essential role as a negative regulator of the Wnt/β-catenin signaling pathway. CK1α phosphorylates β-catenin on S45, which is recognized as a priming event for the subsequent β-catenin phosphorylation by GSK3 on T41, S37, and S33. Complete phosphorylation of β-catenin leads to its proteasomal degradation and attenuation of Wnt signaling (15, 16). This function of CK1α as a negative regulator of Wnt signaling is well described and accepted in the field.

At a time when CK1 paralog-specific inhibitors are entering preclinical, first, and second phase clinical trials (NCT04163718, NCT04243785) (11, 17, 18, 19), it is essential to address another layer of CK1α complexity at the level of physiologically occurring splice variants. This variability may be the source of misleading results, discrepancies, and inconsistencies presented in the literature because different splice variants may differ in their involvement in molecular processes, cell/compartment localization, substrate specificity, or ability to be targeted by the same inhibitor. In this study, we have decided to describe the properties of CK1α, including all its splice variants, and CK1α-like. We have cloned and functionally validated CK1α-like and an updated list of human CK1α splice variants. To this end, we have generated a panel of CK1α/CK1α-like KO cell lines and established rescue assays. We describe the distinct differences in the intracellular localization of individual splice variants and a unique, N-terminus and L-segment dependent, role of CK1α but not CK1α-like, in inhibiting the Wnt/β-catenin pathway.

## Results

### Identification and cloning of splice variants of human CK1α/*CSNK1A1* and CK1α-like/*CSNK1A1L*

NCBI RefSeq database (release 220; last update August 25, 2023) (20) contains four verified transcripts designated as “casein kinase I isoform alpha isoform 1-4.” For the purpose of this study, we have designated them as variants CK1α v1-v4. To complement the list of CK1α splice variants, we used the Genome Aggregation Database - gnomAD (21, 22), which accumulates exome and genome sequencing data from a wide variety of large-scale sequencing projects including Ensembl and GTExPortal. In Table 1, we summarize the nomenclature of transcripts from NCBI RefSeq, gnomAD v2.1.1 based on genomic release GRCh37/hg19 and the latest version of human genome assembly GRCh38/hg38. Interestingly, we did not find the transcript corresponding to the NCBI’s isoform 4 in any of the Ensembl versions.

**Table 1:** The summary of *CSNK1A1* splice variants

Abbreviations: aa, amino acid; CDS, coding sequence; NMD, nonsense mediated decay.

The summary of *CSNK1A1* splice variants in the NCBI RefSeq database and the Ensembl database (for both GRCh37/hg19 as well as GRCh38/hg38 genome assemblies). Splice variants in rows correspond to each other if possible. The “*CSNK1A1*/CK1α” column contains abbreviations for the *CSNK1A1* splice variants and resulting CK1α protein variants used in this study. NMD variants and variants with incomplete CDS were omitted.

The column (gray shaded) contains the nomenclature of CK1α splice variants used in this study. It should enable easier orientation for readers who would like to correlate our nomenclature with database accession numbers.

**Figure 1:**
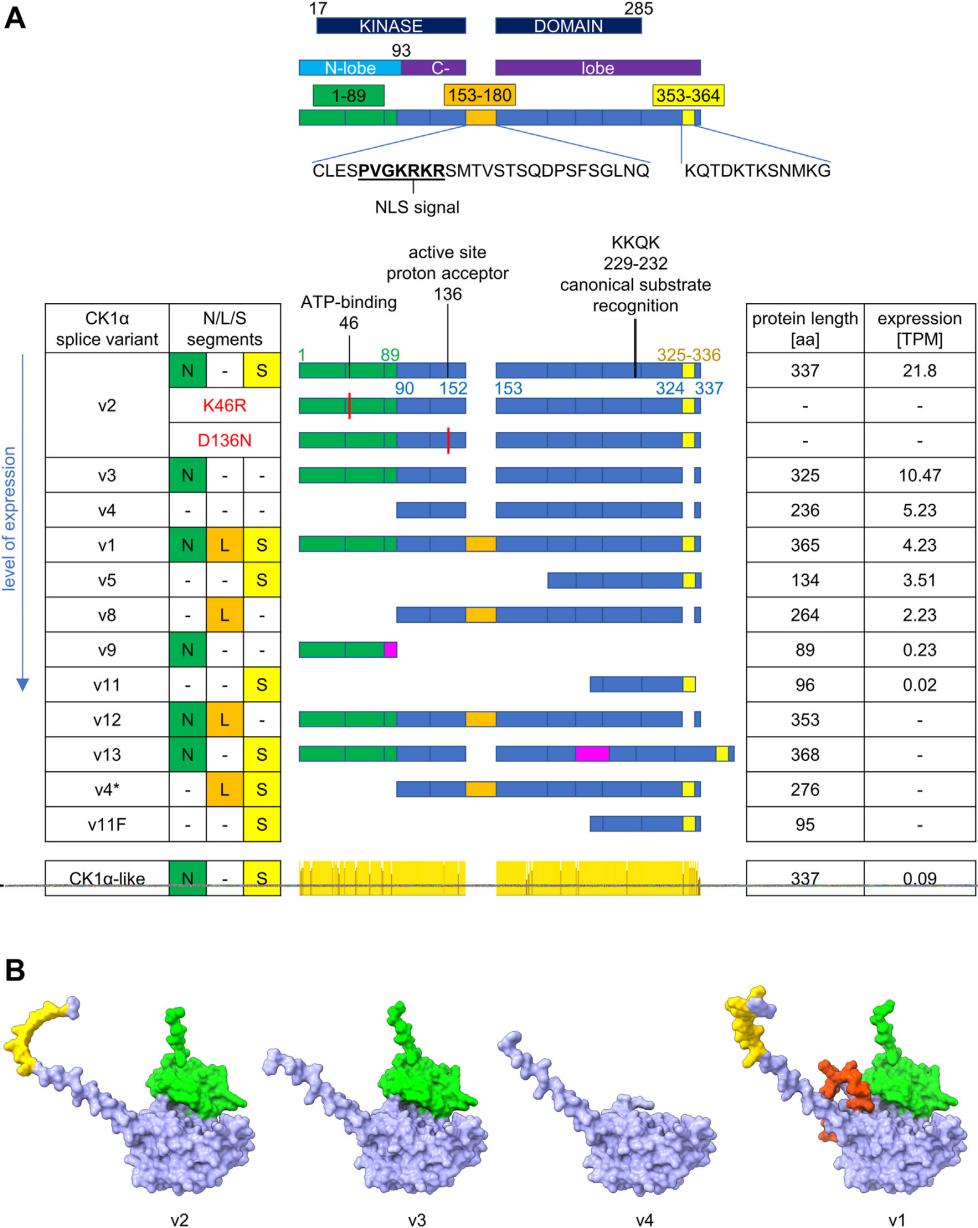
**CK1α splice variants: composition, expression, and structural predictions.***A*, overview of CK1α splice variants and their schematic composition aligned according to the segments. *Green* color represents N-terminal segment, *orange* color represents L-segment, and *yellow* color represents S-segment. *Magenta* color represents unique sections for a given variant. Depiction of CK1α-like reflects conservation of residues between CK1α-v2 and CK1α-like created in Jalview 2.11.2.7 (94). The level of expression represents mean expression across all tissues in TPM (transcripts per million) obtained from gnomAD v2.1.1/GTEx v7 RNA-Seq data for GRCh37/hg19 (last update August 25, 2023) (21, 22). “-” expression data are not available. *B*, AlphaFold2 predictions of four most abundant splice variants (superimposed but visualized separately). N,L,S-segments are highlighted in color code from (*A*). Predictions v2-AF_P48729 and v4-AF_D6REM4 were downloaded from AlphaFold Protein Structure Database (https://alphafold.ebi.ac.uk/) (95, 96). Predictions for v3 and v1 were created by ColabFold (97).

After further quality controls—namely consideration of the nonsense-mediated decay and completeness of the sequence—we ended up with 11 splice variants of CK1α (Table 1), which were further analyzed in this study. Their simplified graphical representation is shown in Figure 1*A*. The protein sequences of each transcript variant were aligned (Fig. S1*A*) and ordered according to their decreasing expression in tissues (Fig. 1*A* and S1*B*). The main source of sequential variability between splice variants is the rearrangement of three segments – N, L, and S, which has significant structural implications as shown by the AlphaFold2 predictions in Figures 1*B* and S1*C*. These variants and *CSNK1A1L*, which is a single exon-encoded gene, were cloned into mammalian expression vectors. We have generated an additional variant, designated here as v11F. Variant v11 is missing one C-terminal amino acid, phenylalanine (F) compared to any other variant (with exception of v9, which is missing substantial portion of the C-terminal part of the kinase). Therefore, a v11F construct, containing this phenylalanine, was prepared to elucidate the function of this sequence change.

### Intracellular localization of CK1α splice variants and CK1α-like

**Figure 2:**
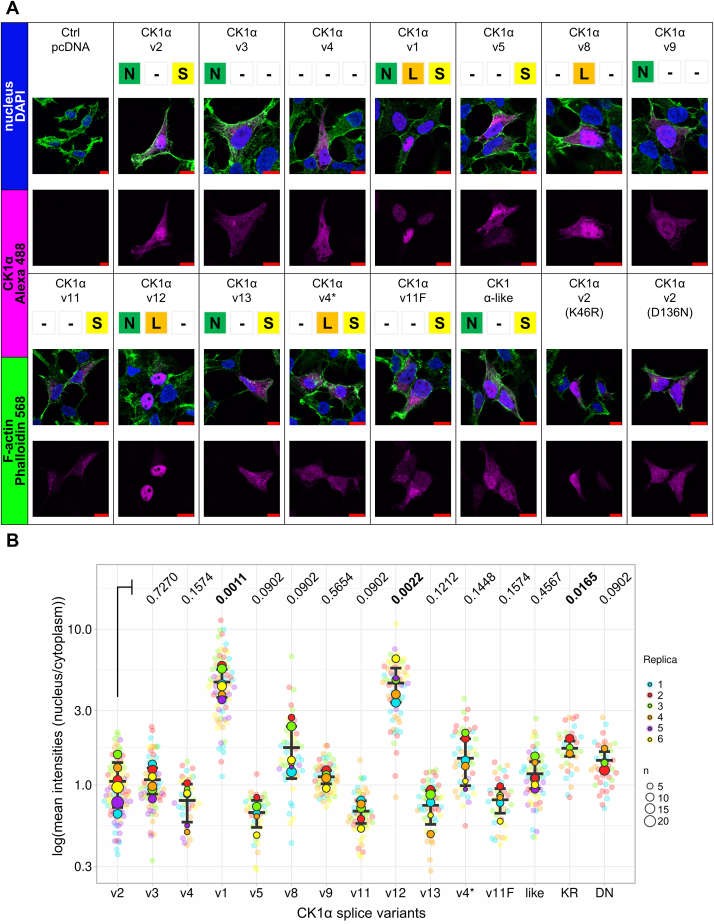
**Analysis of intracellular localization of CK1α splice variants and CK1α-like isoform.***A*, immunocytofluorescence of T-REx-293 cells transfected with vectors coding individual CK1α splice variants and CK1α-like isoform, respectively. Empty pcDNA vector served as the control of staining. Staining was carried out through the N-terminal Flag-tag which is expressed from the vector in fusion with the kinase. Alexa 488 in this case detects Flag-tag. Alexa Fluor 568 Phalloidin detects F-actin. DAPI stains DNA and highlights nuclei. *Red* bars represent 10 μm. *B*, quantification of immunocytofluorescence. Quantification was performed mostly (635/745 images) on multi-channel z-stack tiff images in which layers were processed using sum of pixel values along a single z-axis point. Further, segmentation was performed (whole cell, cytoplasm, nucleus) and the calculation of mean pixel intensities of the regions of interest (nucleus/cytoplasm) was carried out. Graph was created by SuperPlotsOfData web application (https://huygens.science.uva.nl/SuperPlotsOfData/) (85). Statistical analysis: Performed on the means of individual replicates using Brown-Forsythe and Welch ANOVA tests with FDR correction for multiple comparisons (Two-stage step-up method of Benjamini, Krieger, and Yekutieli; Q = 0.05; 14 comparisons; q value is shown). Mean and S.D. error bars. n = 4 to 6 independent biological replicates (1n is on average nine cells).

As a first step, we wanted to analyze the subcellular localization of individual variants of CK1α and CK1α-like. We transfected the expression vectors into T-REx-293 cells and analyzed their subcellular localization by immunocytochemistry. As shown in Figure 2*A* (quantified in Fig. 2*B*), the individual variants showed the full spectrum of subcellular localization patterns, ranging from almost exclusively nuclear to almost exclusively cytoplasmic. It has been previously reported that L-segment is responsible for the nuclear localization of CK1α variants (10). However, our data showed that the combination of N (*i.e.*, intact N lobe of the kinase domain) and L (*i.e.*, segment in the kinase domain, containing the NLS) segments is critical for complete nuclear localization as it is highlighted for v1 and v12 (Fig. 2*B*). Variants v8 and v4^∗^ that also contained L-segment but not the N-segment showed only partial nuclear localization (Fig. 2*B*). Neither the presence nor the absence of the S-segment in these four cases significantly affected kinase localization.

The canonical variant CK1α-v2 showed an even nuclear and cytoplasmic localization, similar to CK1α-like, with which it shares the highest sequence homology (90.5% identity) as well as segment composition. To test if the kinase activity controls the subcellular localization of CK1α-v2, we have prepared mutant K46R (KR) of ATP-binding site of CK1α-v2 and mutant D136N (DN) of the catalytic proton acceptor site of CK1α-v2, which were previously described to impair kinase activity (23, 24, 25, 26). Interestingly, these kinase activity–compromised mutants showed a tendency towards more nuclear localization, which was in the case of K46R significant (Fig. 2*B*). On the other hand, variants lacking the N-segment and thus part of the N-terminal lobe (v5, v11, and v11F) shifted to a more cytoplasmic localization. In conclusion, these experiments showed that although individual N, L, and S segments affect subcellular localization of CK1α, only their combination together with the kinase activity is sufficient to fully determine the nucleo-cytoplasmic distribution of CK1α.

### Derivation and characterization of cells deficient in CK1α/*CSNK1A1*, CK1α-like/*CSNK1A1L*, and both

**Figure 3:**
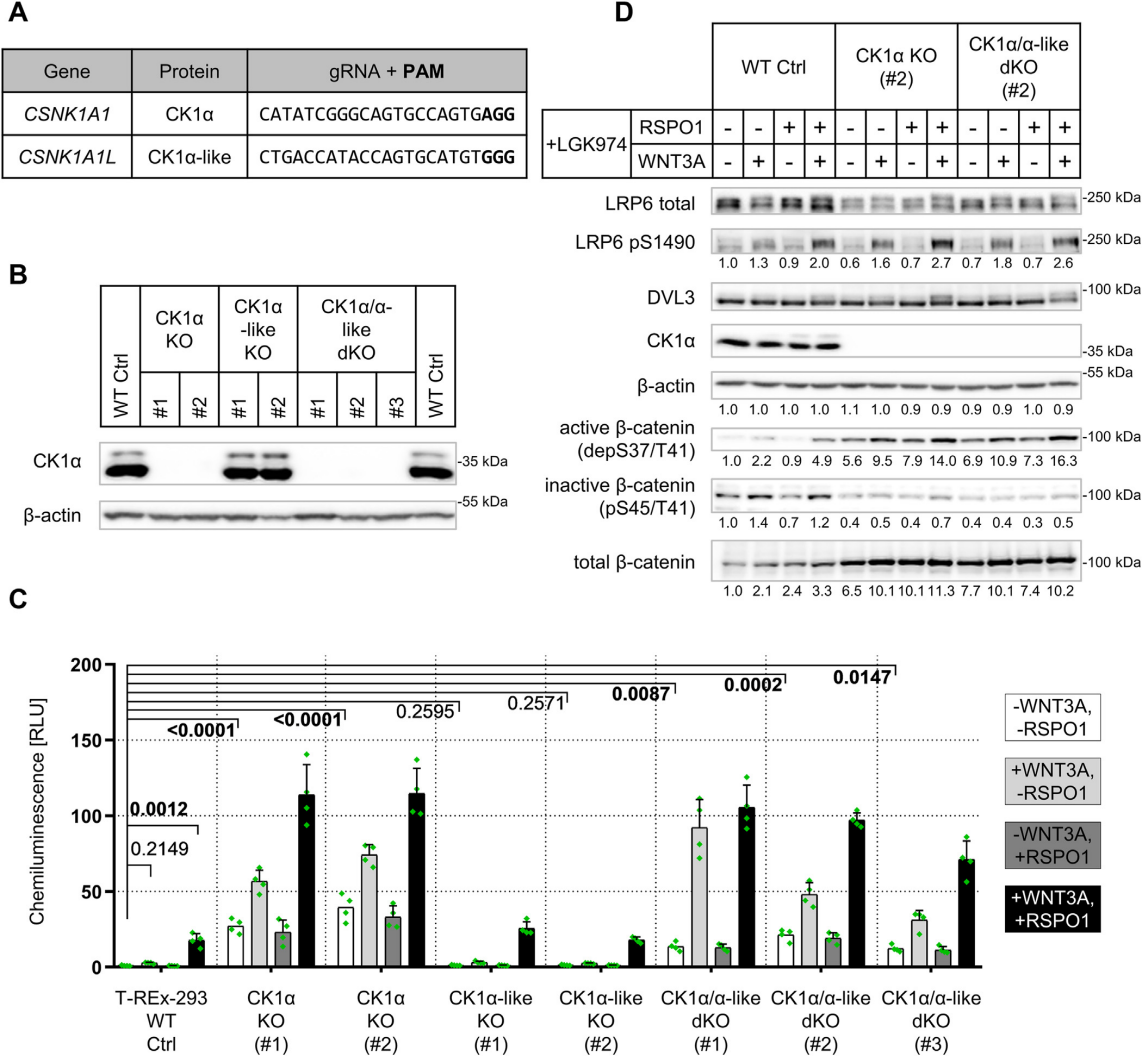
**Production, validation and functional characterization of CK1α/α-like KO panel cell lines.***A*, gRNA sequences used to produce single and compound KO lines of CK1α/α-like KO panel (*B*). PAM is highlighted in *bold* and is not part of the gRNA sequence. *B*, Western blot validation of single and compound KO lines on protein level. *C*, TopFlash analysis of CK1α/α-like KO panel lines treated for 16 h in combinations with Wnt-3a (WNT3A) recombinant protein (80 ng/ml) and/or R-spondin-1 (RSPO1) recombinant protein (25 ng/ml). Treatment with LGK974 (0.5 μM) in all conditions. Normalized ratios between TopFlash luciferase and Renilla luciferase are plotted. Statistical analysis: Two-Way ANOVA with FDR correction for multiple comparisons (Two-stage step-up method of Benjamini, Krieger, and Yekutieli; Q = 0.05; 496 comparisons; q value is shown). Mean and S.D. error bars. n = 4 independent biological replicates. *D*, Western blot analysis of CK1α KO (#2) and CK1α/α-like dKO (#2) lines using protein lysate from TopFlash analysis. Numbers under the blots represent relative densitometry values normalized to a loading control (β-actin). RLU, relative luminescence units.

CK1α may have both negative and positive roles in controlling the Wnt/β-catenin pathway (15, 16, 27, 28). Nothing is known about the function of CK1α-like and its possible redundancy with CK1α. To clarify the role of CK1α and CK1α-like in the Wnt/β-catenin pathway, we took advantage of the CRISPR/Cas9 technology and established a panel of T-REx-293 cell lines depleted of the endogenous CK1α, CK1α-like, and both (Fig. 3*A*). Multiple clones were isolated for each type of KO line, and monoclonal cell lines were established. The cell lines were thoroughly validated by restriction fragment length polymorphism, Western blotting (WB), and next generation sequencing (Figs. 3*B* and S2). WB analysis was performed with a commercially available CK1α-specific antibody raised against a synthetic peptide containing human CK1α-v2 amino acids 50-150aa. The commercial CK1α-like antibody was also tested, but it showed cross-reactivity with CK1α (data not shown). Two clones for each single KO line and three clones for CK1α/CK1α-like double KO (dKO) line together with the WT T-REx-293 cells as a control (WT Ctrl) were selected and characterized for their ability to transduce Wnt/β-catenin signaling.

The activity of the Wnt/β-catenin pathway was analyzed using the reporter system known as TopFlash (29), where increased TCF/LEF-dependent transcription translates into increased activity of firefly luciferase. All cells were pretreated with the porcupine inhibitor LGK974 (30), which blocks secretion of Wnt ligands and prevents any autocrine activation. The Wnt/β-catenin pathway activity was studied using four conditions: (i) no stimulation, (ii) stimulation with Wnt-3a, a prototypical ligand of the Wnt/β-catenin signaling (31), (iii) stimulation with R-spondin-1, which prevents internalization of FZD receptors (32), as well as (iv) with the combination of Wnt-3a and R-spondin-1 for maximal pathway stimulation. As shown in Figure 3*C*, the lack of CK1α resulted in the augmented Wnt/β-catenin signaling both in the basal and activated conditions; CK1α-like KOs were comparable to WT, and CK1α/CK1α-like dKOs had a similar effect as CK1α KOs. This phenotype was remarkably stable across multiple clones examined. These data suggested that endogenous CK1α was a potent negative regulator of the Wnt/β-catenin pathway (in agreement with earlier studies by Amit *et al.* (15); Liu *et al.* (16)), whereas endogenous CK1α-like has no obvious role in regulating Wnt signaling. Interestingly, Wnt-3a (with or without R-spondin-1) still significantly activated TopFlash in both CK1α KO and CK1α/CK1α-like dKO cells, suggesting that (i) the loss of CK1α does not lead to maximal activation of the pathway and (ii) the absence of CK1α does not negatively affect the proximal ligand-receptor molecular machinery.

We then performed a detailed Western blot analysis of Wnt/β-catenin pathway readouts using two clones, CK1α KO (#2) and CK1α/α-like dKO (#2). We analyzed the phosphorylation of LRP6, DVL3, and β-catenin, which was detected either with a phosphorylation-specific antibody or as an electrophoretic mobility up-shift (hereafter referred to as a shift) (Fig. 3*D*). As expected, cells lacking CK1α showed higher β-catenin levels, due to the accumulation of active (non-phospho S37/T41) β-catenin and the reduction of inactive and degradation-prone pS45/T41-β-catenin. We did not observe any deficit in the activation of LRP6 and phosphorylation of DVL3. Taken together, our analysis of different CK1α KO cells suggests that CK1α (but not CK1α-like) in a ligand-independent manner negatively regulates the Wnt/β-catenin pathway at the level of β-catenin phosphorylation and degradation.

### Characterization of CK1α splice variants and CK1α-like in rescue assays

The sensitization and hyperactivation of the Wnt signaling pathway in cells lacking CK1α represents an ideal readout for a rescue assay. Therefore, we used CK1α/CK1α-like dKO cells to test the ability of individual CK1α splice variants and CK1α-like to act as the negative regulator of the Wnt/β-catenin pathway. For this purpose, we re-expressed CK1α/CK1α-like variants in WT and CK1α/α-like dKO (#2) line and analyzed TopFlash activity under full (Wnt-3a/R-spondin-1) or no stimulation.

**Figure 4:**
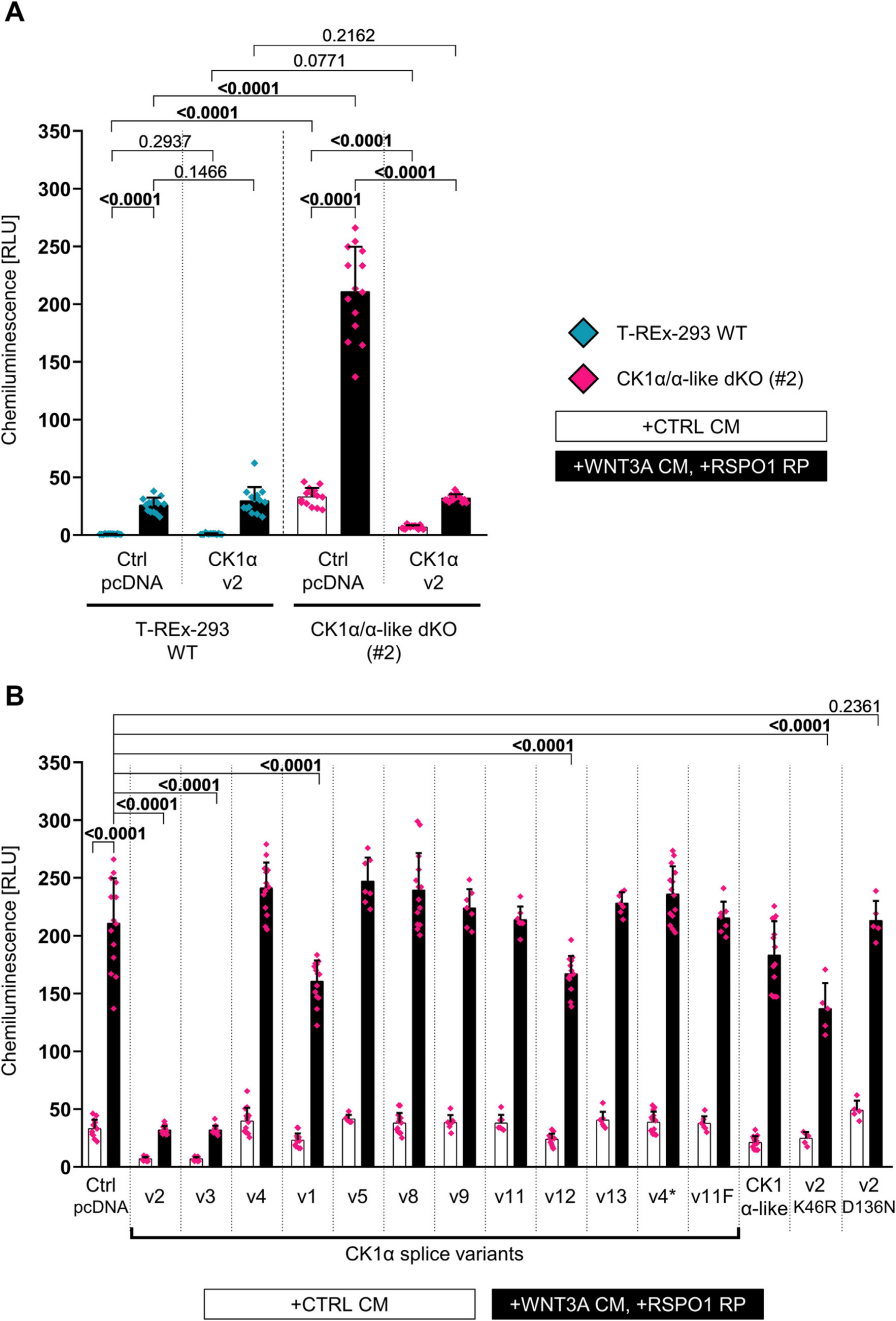
**Evaluation of CK1α splice variants and CK1α-like activity in the Wnt/β-catenin pathway through functional rescue assay.** TopFlash rescue assay was performed on WT cells (*A*, *blue* data points) as well as CK1α/α-like dKO (#2) cells (*A* and *B*; *pink* data points). The amount of transfected vector expressing splice variant was 50 ng. pcDNA condition served as the control of transfection. All conditions were pretreated with porcupine inhibitor LGK974 (0.5 μM) to set basal state. Cells were further stimulated with Wnt-3a conditioned medium (WNT3A CM) with the addition of R-spondin-1 recombinant protein (RSPO1 RP) (25 ng/ml) for 16 h. The control conditions were treated with control conditioned medium (CTRL CM). Normalized ratios between TopFlash luciferase and Renilla luciferase are plotted. Statistical analysis: Outliers were identified through ROUT method (Q = 1%) and excluded (4/684 measurements). Cleaned data were analyzed through Three-Way ANOVA with FDR correction for multiple comparisons (Two-stage step-up method of Benjamini, Krieger, and Yekutieli; Q = 0.05; 2016 comparisons; q value is shown). Mean and S.D. error bars. n = 5 to 14 independent biological replicates. *A*, graph highlights the importance of CK1α/α-like dKO model line system for the evaluation of activity of CK1α splice variants. *B*, graph highlights the conditions of rescue experiment where CK1α/α-like dKO (#2) cells are transfected with expression vectors for individual CK1α splice variants, CK1α-like isoform, or kinase dead mutants (K46R and D136N) of canonical variant CK1α-v2. RLU, relative luminescence units.

Expression of CK1α/CK1α-like in WT cells did not show any phenotype (Figs. 4*A* and S3*A*) despite significant expression of all used constructs (Fig. S3*B*). This effect is most likely due to the functional saturation by the endogenous CK1α. On the contrary, re-expression of CK1α-v2 efficiently downregulated the hyperactivated TopFlash in CK1α/α-like dKO cells, both in the presence and absence of external stimulation (Fig. 4*A*). We then used this rescue assay to test the full panel of CK1α/CK1α-like variants (Fig. 4*B*). Surprisingly, only two variants—CK1α-v2 and v3—were able to fully rescue and act as potent negative regulators. Partial, albeit very limited rescue, was also observed for other variants containing the full kinase domain—namely nuclear CK1α variants v1 and v12, CK1α-v2(K46R), and CK1α-like. Interestingly, CK1α-v13 which is identical to CK1α-v2 except for a 31aa insertion of a unique sequence in the kinase domain was functionally completely inactive, most likely due to the steric blocking of the substrate recognition sequence caused by the insertion (see Figs. S1*A* and 1*C*).

The rescue assay enabled us to further test several questions that had arisen after the initial screening. More specifically, we addressed the following questions: (i) Is the nuclear localization of v1 and v12 the reason for their inactivity in the Wnt/β-catenin pathway? (ii) Can variants encoding the N- and C-terminal portions of CK1α complement each other to form a functional kinase? (iii) Can variants encoding the C-terminal portions of CK1α act as negative regulators of the full-length CK1α *via* a decoy mechanism?

**Figure 5:**
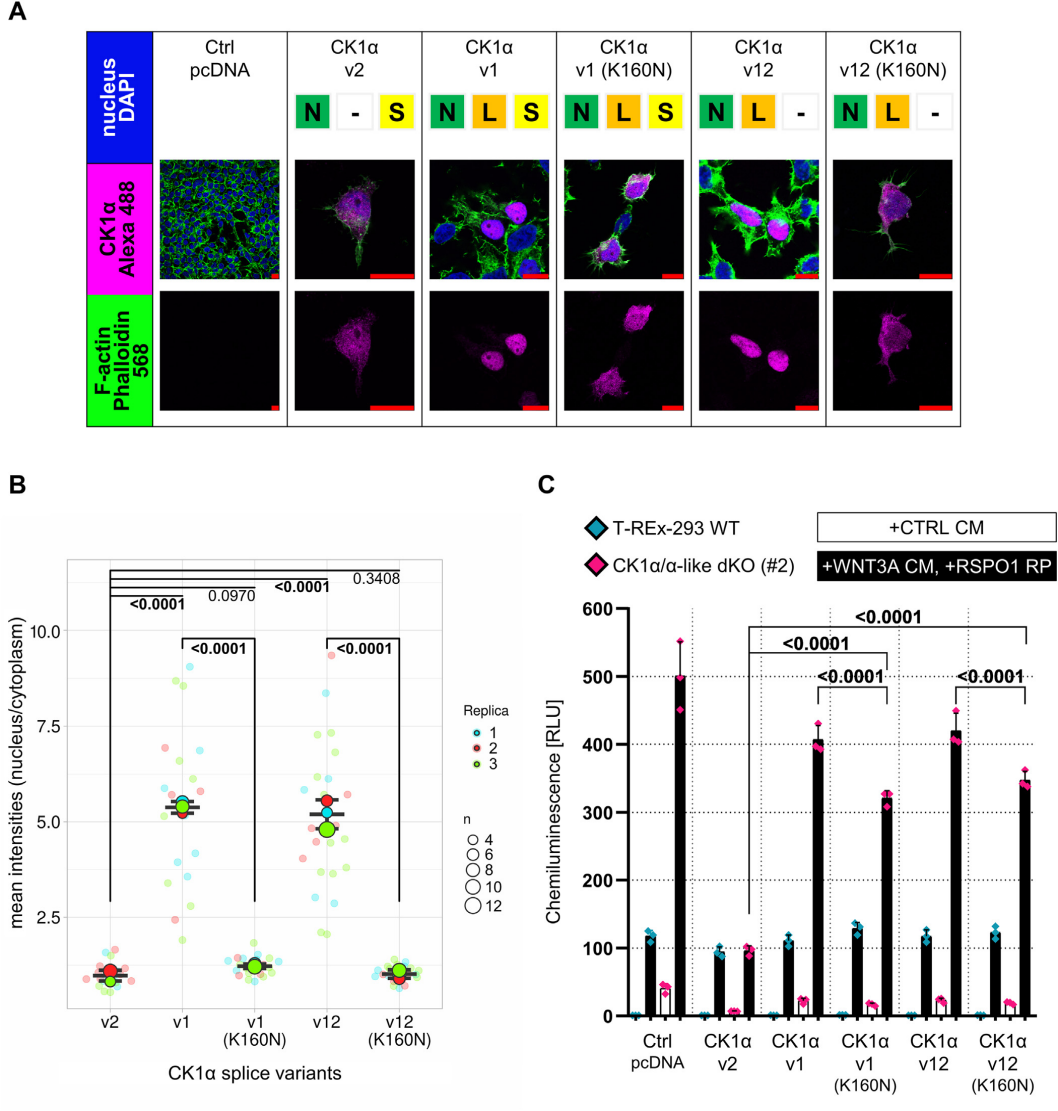
**Impact of NLS mutation on subcellular localization and activity of CK1α splice variants.***A*, immunocytofluorescence of T-REx-293 cells transfected with vectors coding CK1α splice variants v1, v12, and their NLS mutants (K160N), respectively. Empty pcDNA vector and variant v2 served as controls of staining and localization, respectively. Staining was carried out through the N-terminal Flag-tag which is expressed from the vector in fusion with the kinase. Alexa 488 in this case detects Flag-tag. Alexa Fluor 568 Phalloidin detects F-actin. DAPI stains DNA and highlights nuclei. *Red* bars represent 10 μm. *B*, quantification of immunocytofluorescence. Quantification was performed on multi-channel z-stack tiff images (107 images) in which layers were processed using sum of pixel values along a single z-axis point. Further, segmentation was performed (whole cell, cytoplasm, nucleus) and the calculation of mean pixel intensities of the regions of interest (nucleus/cytoplasm) was carried out. Graph was created by SuperPlotsOfData web application (https://huygens.science.uva.nl/SuperPlotsOfData/) (85). Statistical analysis: Performed on the means of individual replicates using Ordinary One-way ANOVA with FDR correction for multiple comparisons (Two-stage step-up method of Benjamini, Krieger, and Yekutieli; Q = 0.05; 10 comparisons; q value is shown). Mean and S.D. error bars. n = 3 independent biological replicates (1n is on average seven cells). *C*, TopFlash assay - rescue assay with CK1α splice variants v1, v12, and their NLS mutants (K160N), respectively. Empty pcDNA vector and variant v2 served as controls of transfection and activity, respectively. The assay was performed on WT cells (*blue* data points) as well as CK1α/α-like dKO (#2) cells (*pink* data points). The amount of transfected vector was 50 ng. Cells were stimulated with Wnt-3a conditioned medium (WNT3A CM) with the addition of R-spondin-1 recombinant protein (RSPO1 RP) (25 ng/ml) for 16 h. The control conditions were treated with control conditioned medium (CTRL CM). All conditions were pretreated with porcupine inhibitor LGK974 (0.5 μM) to set basal state. Normalized ratios between TopFlash luciferase and Renilla luciferase are plotted. Statistical analysis: Three-Way ANOVA with FDR correction for multiple comparisons (Two-stage step-up method of Benjamini, Krieger, and Yekutieli; Q = 0.05; 276 comparisons; q value is shown). Mean and S.D. error bars. n = 3 independent biological replicates. RLU, relative luminescence units.
(ii) Splice variants giving rise to the N-terminal (v9) and C-terminal portions (v4, v8, and v4^∗^) of CK1α could theoretically, based on their sequences and AlphaFold2 predictions (data not shown), form a reconstituted N-lobe and C-lobe of the kinase domain. To test this possibility, we cotransfected these variants (v9 with v4, v8, and v4^∗^, respectively). As shown in Fig. S3*C*, none of these combinations functionally complemented. We therefore concluded that such complementation is unlikely to occur in cells.
(iii) CK1 kinases (33, 34, 35) and CK1α in particular (36) can be regulated by inhibitory autophosphorylation at their C-termini. CK1α splice variants harboring the C-terminus (v4, v5, v11) could therefore act as pseudo-substrates and inhibit the activity of other splice variants (v2, v3, v1, v12). We tested this possibility in the rescue assay (Fig. S3*D*). However, we did not observe any effects in the cotransfections with any of the fully active variants (v2 nor v3). However, the v4 and v5 (but not v11) variants reversed the partial rescue of CK1α-v1 and v12 and a positive correlation between the length of the variants (v4, v5, v11) and the level of activation was observed. The difference between v2/v3 and v1/v12 phenotypes could be related to the change in the substrate specificity between the two isoforms which will be discussed in the next chapter.

(i) We prepared CK1α-v1(K160N) and CK1α-v12(K160N) harboring the mutation in the NLS within the L-segment (Figs. 1*A* and S1*A*) (10). As shown in Figure 5*A* and quantified in Figure 5*B*, mutation of the NLS resulted in the reversal of the nuclear localization to an evenly distributed nuclear and cytoplasmic localization, characteristic of splice variant v2. Interestingly, such complete switch in the subcellular localization was accompanied by only partial restoration of the activity in the rescue assay (Fig. 5*C*). This suggests that the localization of the exclusively nuclear CK1α-v1 and v12 cannot fully explain the observed effect seen on pathway regulation. We therefore propose that the presence of the L-segment in the kinase domain also alters other properties of CK1α, possibly the interaction with regulatory proteins or its substrate specificity.

### CK1α splice variants and CK1α-like can phosphorylate β-catenin *in vitro*

**Figure 6:**
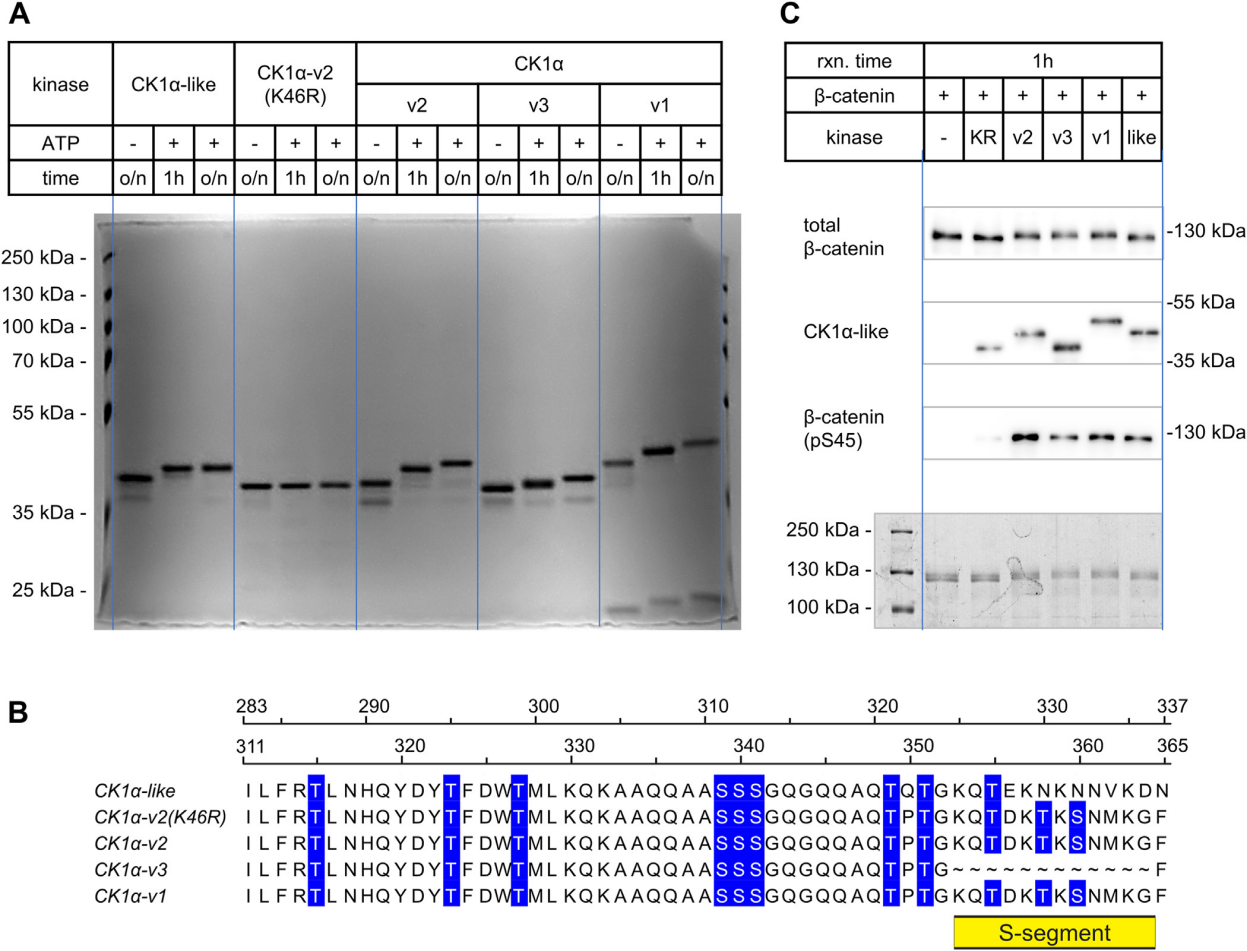
**Assessment of CK1α splice variants and CK1α-like kinase activity*****in vitro*****.***A*, *in vitro* kinase assay (IVKA) – Coomassie Blue R-250 stained 12% acrylamide gel showing autophosphorylation assay performed using 1 μg of 6xHis-CK1α-like, 6xHis-CK1α-v2(K46R) kinase dead mutant as a control and 6xHis-CK1α variants v2, v3, v1, respectively. Reaction was carried out at two time points (1 h, overnight) in the presence of ATP (1 mM) and o/n in the absence of ATP as a control. MgCl_2_ (10 mM) and EDTA (1 mM) were present. *B*, multiple protein sequence alignment of proteins from (*A*). Serines (S) and threonines (T) are colored in *blue*. The span of S-segment is highlighted. Alignment was created in Jalview 2.11.2.7 (94). The alignment ruler reflecting protein length is visualized: the top one is for CK1α-like/-v2(K46R)/-v2/-v3 and the bottom one is for CK1α-v1. *C*, IVKA between 6xHis-GST-FL-β-catenin as a substrate and 6xHis-CK1α splice variants (v2, v3, v1), as well as 6xHis-CK1α-like. Reaction with just substrate without kinase (−), as well as 6xHis-CK1α-v2(K46R) kinase dead mutant served as the controls. Depicted are immunoblots and Coomassie Blue R-250 stained 12% acrylamide gel providing a loading control.

In the light of our results, we decided to test the kinase autophosphorylation activity of CK1α/CK1α-like variants in kinase assays. We purified the three most expressed CK1α splice variants containing a complete kinase domain (v2, v3, and v1), the CK1α-like and CK1α-v2(K46R), a kinase-dead variant serving as a negative control. First, we tested the kinase activity of the purified kinases using the autophosphorylation assay (Fig. 6*A*). Using a phosphorylation-dependent mobility shift, we were able to show that all tested kinases, except for the kinase-dead variant CK1α-v2(K46R), were efficiently autophosphorylated. The mobility shift was less pronounced for CK1α-v3, which is likely due to the loss of T327, T330, and S332, three potential autophosphorylation sites, located in the S-segment (Fig. 6*B*). Next, we used full-length β-catenin as a substrate. Phosphorylation of β-catenin at S45 was analyzed by WB. As shown in Figure 6*C*, all four kinases, CK1α-v2, v3, v1, and CK1α-like, did phosphorylate S45 of β-catenin with comparable efficiency. Unexpectedly, we repeatedly observed residual activity of CK1α-v2(K46R) mutant on the phosphorylation of S45 of β-catenin. This observation is in discrepancy with the literature (15, 23, 24, 25, 26); however, it supports our results in Figure 4*B* showing slight but significant negative effect of this mutant in the rescue assay. This experiment demonstrated that all four kinases have kinase activity and can phosphorylate β-catenin. We believe that these data are an important finding, especially with respect to CK1α-like, for which no catalytic activity has been reported so far.

### Endogenous CK1α and GSK3α/β are required for the full phosphorylation of Axin

**Figure 7:**
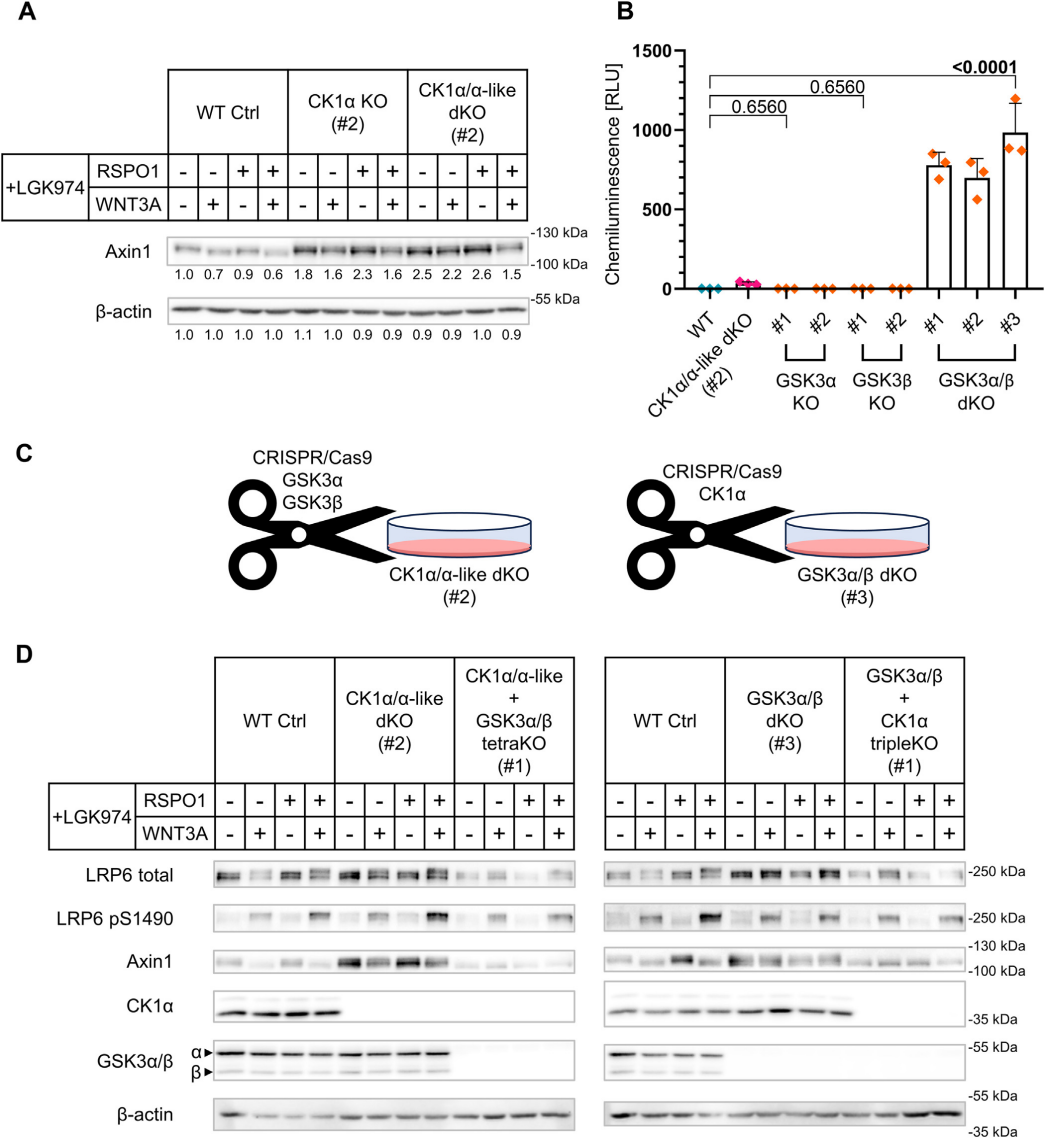
**CK1α depletion causes Axin1 accumulation and prevents Wnt-3a-induced Axin1 dephosphorylation.***A*, the extension of Figure 3*D*. Western blot analysis of CK1α KO (#2) and CK1α/α-like dKO (#2) lines exhibiting phenotype on Axin1. Protein lysates from TopFlash analysis (Fig. 3*C*) were used. Blot showing β-actin is identical to the one in Figure 3*D*. Numbers under the blots represent relative densitometry values normalized to a loading control (β-actin). *B*, TopFlash analysis of GSK3α/β KO panel (*orange* data points): Only GSK3α/β dKO clones show upregulated Wnt/β-catenin signaling. Cells were treated for 16 h with control conditioned medium (CTRL CM) and LGK974 (0.5 μM). Normalized ratios between TopFlash luciferase and Renilla luciferase are plotted. Statistical analysis: Ordinary one-way ANOVA with FDR correction for multiple comparisons (Two-stage step-up method of Benjamini, Krieger, and Yekutieli; Q = 0.05; eight comparisons with WT condition; q value is shown). Mean and S.D. error bars. n = 3 independent biological replicates. *C*, depiction showing the generation of CK1α/α-like + GSK3α/β tetraKO cells on the background of CK1α/α-like dKO (#2) cells as well as GSK3α/β + CK1α tripleKO cells on the background of GSK3α/β dKO (#3) cells. *D*, Western blot analysis of WT, CK1α/α-like dKO (#2), GSK3α/β dKO (#3), CK1α/α-like + GSK3α/β tetraKO (#1), and GSK3α/β + CK1α tripleKO (#1) cells treated for 16 h in combinations with Wnt-3a conditioned medium (WNT3A CM) or control conditioned medium (CTRL CM) together with or without R-spondin-1 recombinant protein (RSPO1 RP) (25 ng/ml). All conditions were treated with porcupine inhibitor LGK974 (0.5 μM) to set basal state. Phosphorylation status of Axin can be monitored by the phosphorylation mobility shift. RLU, relative luminescence units.

The combination of results of the rescue assay (Fig. 4), in which only CK1α-v2 and v3 showed full activity, and *in vitro* kinase assay (Fig. 6) where all tested variants (CK1α-v2, v3, v1, and CK1α-like) phosphorylated S45 of β-catenin suggests that the role of CK1α in the Wnt/β-catenin signaling pathway is more complex than just phosphorylation of S45 of β-catenin. In order to address this question, we have reanalyzed our CK1α/α-like KO panel of cell lines (introduced in Fig. 3*D*) and observed a clear phenotype on Axin1. Axin1 is a key regulatory scaffold protein of the canonical Wnt pathway, which forms the destruction complex together with adenomatous polyposis coli (APC), CK1α, and GSK3α/β (for review, see (37, 38)). Wnt-3a triggers inactivation of the destruction complex that is accompanied by Axin dephosphorylation and degradation (39, 40, 41, 42). We could fully reproduce these Wnt-3a effects in our experimental system (Fig. 7*A*) where endogenous Axin1 downshifts (dephosphorylation) and decreases on intensity on Western blotting. Interestingly, depletion of CK1α caused accumulation of Axin1 and eliminated Wnt-3a–induced dephosphorylation (Fig. 7*A*). While accumulation can be caused by the compensatory mechanism in conditions with the overactivated Wnt pathway, the absence of Wnt-3a–induced dephosphorylation clearly suggests that CK1α is required for this process.

The phosphorylation of Axin is an important regulatory step in destruction complex function. It has been proposed already by Luo *et al.* (43) that similarly to β-catenin, the full phosphorylation of Axin1 is achieved by GSK3 and CK1; upon Wnt stimulation, Axin1 gets dephosphorylated by protein phosphatase 1 (PP1). In order to test the role of GSK3, we have prepared GSK3α/β KO panel (Fig. S4, *A* and *B*) to compare the phenotypes of KO lines in our model cell lines. We observed redundancy of GSK3α and GSK3β isoforms described earlier (44) and also confirmed the well-known fact that deletion of GSK3 dramatically increases Wnt/β-catenin signaling (44) and this is to a much higher extent than in CK1α KO (Fig. 7*B*). Finally, to study the combined activity of CK1α and GSK3α/β, we have prepared the compound KO lines. We have used two independent approaches and prepared CK1α/α-like + GSK3α/β tetraKO line on the background of CK1α/α-like dKO (#2) line as well as GSK3α/β + CK1α tripleKO line on the background of GSK3α/β dKO (#3) line (Fig. 7*C*). These cells retained highly active Wnt/β-catenin signaling in the absence of Wnt (Fig. S4*C*).

With respect to Axin1, the deletion of GSK3α/β did not result in a full Axin1 dephosphorylation, but GSK3α/β KO cells clearly failed to respond to Wnt-3a both at the level of Axin1 dephosphorylation and degradation (Fig. 7*D*). Importantly, the combined depletion of CK1α and GSK3α/β resulted in a phenotype of completely dephosphorylated Axin1, mimicking in this respect the full activation of the pathway induced by Wnt-3a (Figs. 7*D* and S4*D*). We conclude that despite both GSK3 and CK1α can independently phosphorylate Axin1, only their combined activity leads to the fully (or qualitatively differently) phosphorylated and hence active Axin1. Only Axin1 phosphorylated in this way can be recognized by PP1 after Wnt-3a stimulation in order to be dephosphorylated. Our data thus provide genetic proof of mechanism proposed by Luo *et al.* (43) and demonstrate that CK1α and GSK3 in the destruction complex cooperate not only to achieve complete degradation of β-catenin but also full phosphorylation of Axin1.

### CK1α splice variants but not CK1α-like are potently inhibited by ATP-competitive inhibitors

**Figure 8:**
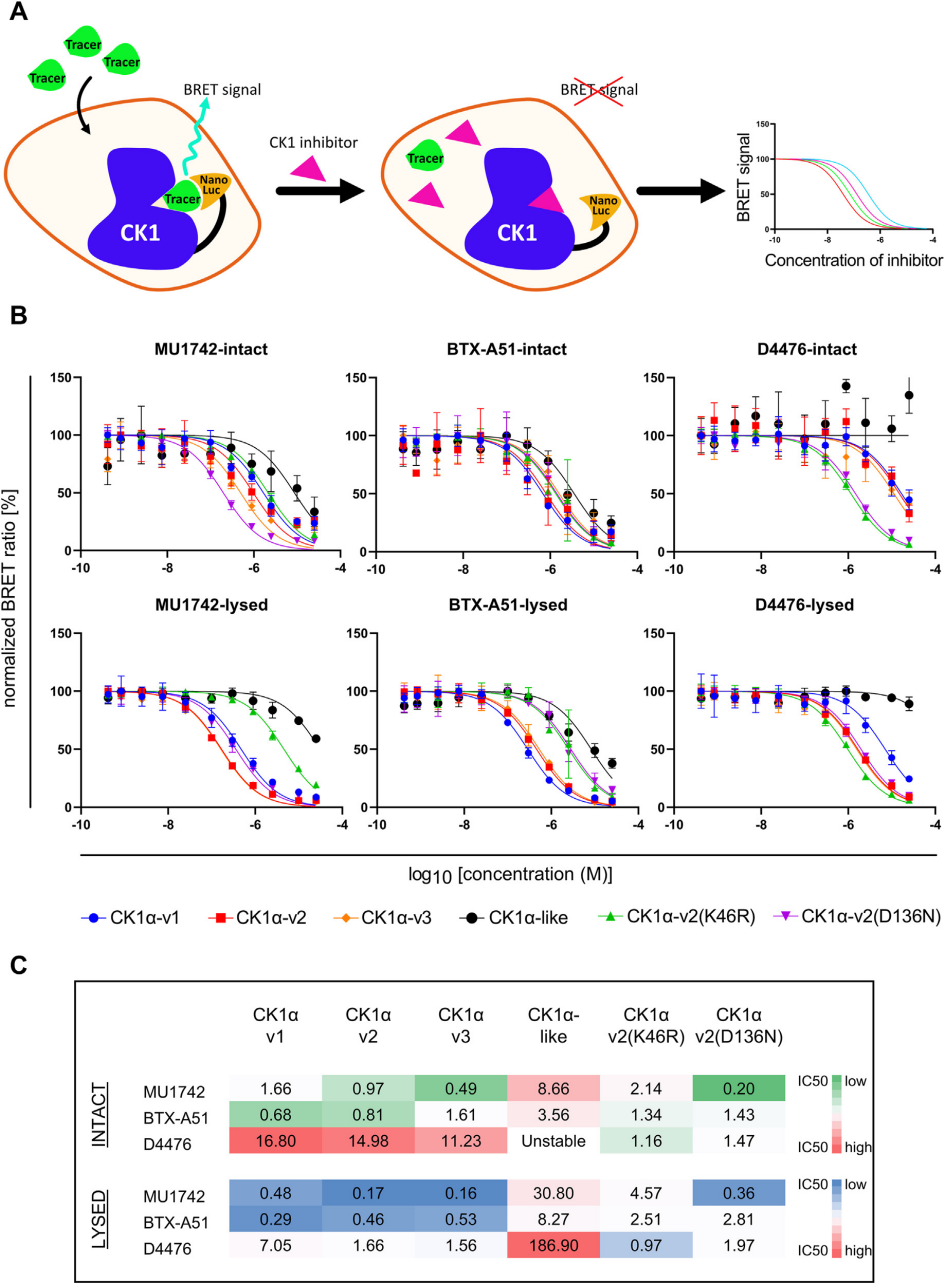
**Quantitative assessment of CK1α inhibitor binding using NanoBRET target engagement assay.***A*, scheme of the principle of NanoBRET target engagement assay. In the absence of CK1 inhibitor, energy transfer from the NanoLuc tag of CK1 to the Tracer takes place resulting in the emission of BRET signal. Addition and binding of CK1 inhibitor displaces the Tracer resulting in the quantitative disappearance of BRET signal. *B*, NanoBRET target engagement assay curves in intact cells and in lysed cells as a control - characterization of potency of selected inhibitors (MU1742, BTX-A51, D4476) against CK1α splice variants and CK1α-like isoform. n = 3 to 4 independent biological replicates. *C*, table showing calculated IC50 values (μM) from NanoBRET graphs in (*B*). BRET, Bioluminescence Resonance Energy Transfer.

In the final part of our study, we focused on characterizing CK1α splice variants and CK1α-like with respect to their ability to be inhibited by CK1 inhibitors. To study cellular target engagement, we developed nanoBRET tracer displacement assays (Fig. 8*A*), which allows for quantitative assessment of compound binding to target proteins in living cells. The method is based on Bioluminescence Resonance Energy Transfer (BRET) from the NanoLuc-luciferase–tagged CK1α (energy donor) to the cell-permeable fluorescent tracer (energy acceptor). The tracer binds reversibly to the ATP-binding pocket of CK1α. The tracer is displaced upon inhibitor titration resulting in a reduction of the BRET signal (45, 46).

We tested CK1α isoforms and mutants (v2, v3, v1, v2(K46R), v2(D136N)) and CK1α-like in the developed BRET assays. The compounds tested were MU1742, BTX-A51, and D4476. D4476 is an inhibitor of CK1δ and ALK5 (47); however, it has been shown to block CK1α at high concentrations (48, 49, 50). It has been used in the past for the description of the CK1α biology, mainly due to the lack of more CK1α-specific inhibitors at that time. BTX-A51 is the first inhibitor targeting CK1α to enter clinical trials (NCT04243785) for the treatment of acute myeloid leukemia and myelodysplastic syndrome (11, 17, 18). MU1742 is a recently developed small molecule inhibitor of CK1α with kinome-wide selectivity superior to that of BTX-A51 (50).

NanoBRET measurements were performed in two modes using intact and lysed cells to account for potential problems of cell penetration of the tested small molecules (Fig. 8*B*). Our results (summarized in Fig. 8*C*) showed that all splice variants bound the selected inhibitors with comparable potency. In contrast, the tested inhibitors target CK1α-like with much lower affinity. We conclude that (i) the established NanoBRET assay was functional and constant with other cellular assays reporting inhibition of CK1α (50) and (ii) individual CK1α variants are targeted with equal potency suggesting that ATP-competitive CK1α inhibitors target all CK1α splice variants but not CK1α-like *in vivo*.

## Discussion

In our study, we generated cell lines lacking CK1α, CK1α-like, or both. The key phenotype was a greatly increased TCF/LEF-dependent transcription in cells lacking CK1α. The phenotype was very consistent among the clones, demonstrating that no other CK1 paralog could replace CK1α as a negative regulator of the Wnt/β-catenin pathway. This observation was in agreement with multiple studies showing that CK1α-mediated phosphorylation on Ser45 of the β-catenin is required for proper function of the destruction complex (15, 16). However, we very consistently demonstrated that Wnt-3a treatment can further increase the TCF/LEF-dependent transcription in CK1α KO cells (Fig. 3*C*). These data are in line with other studies showing that CK1α is not absolutely required to prime GSK3-mediated phosphorylation of β-catenin on the T41, S37, and S33 cluster (51, 52, 53, 54). Our data allows the combination of both mechanisms and suggest the existence of two levels of β-catenin regulation. The first level is a Wnt-independent, but CK1α-dependent process that controls the basal efficacy of β-catenin degradation and downstream Wnt signaling. The second level is CK1α-independent, but Wnt-dependent and it is likely caused by an additional mechanism of GSK-3β inactivation in the presence of Wnt as previously proposed (55, 56, 57, 58, 59). Thus, only the combination of both processes (*i.e.*, depletion/inhibition of CK1α together with Wnt-3a stimulation) fully unleashes β-catenin resulting in maximal downstream signaling.

We took advantage of the CK1α KO model to establish a rescue assay. Re-introduction of CK1α completely rescued the phenotype of CK1α KO but interestingly did not block Wnt-3a–induced activation or further downregulated TopFlash activity in WT cells. These data support the suggestion in the previous paragraph that there is a CK1α-independent mechanism for Wnt-3a–induced activation of Wnt/β-catenin signaling that cannot be attenuated or blocked by overexpression of CK1α. In addition, overexpressed CK1α cannot further reduce the “basal” activation of Wnt/β-catenin signaling, suggesting that, at this level of regulation, the system is already fully saturated by the activity of endogenous CK1α.

The rescue assay allowed us to functionally test multiple splice variants of CK1α reported in human and other species. Given the inconsistent nomenclature, we have summarized all published evidence across individual species in Table 2 and Fig. S5. The variability, particularly with respect to the use of L and S segments, is present across species, which suggests that CK1α diversity is evolutionary conserved. While previous studies suggested the existence of splice variants, it was Fu *et al.* (10) who first conducted a comprehensive study on the chicken CK1α splice variants and demonstrated the importance of the L-segment, which contains the NLS, for nuclear localization. Our findings expand this study by demonstrating that the L-segment alone is insufficient for the nuclear localization (*e.g.*, v8, v4^∗^) but that only a combination of the N- and the L-segment results in complete CK1α nuclear translocation (*e.g.*, v1, v12). We speculate that the N-segment may serve as an interaction surface, primarily for stable nuclear import complexes to form.

**Table 2:** The literature review on *CSNK1A1* splice variants

Publication	Model	Present segments				Notes
		N--	N-S	NL-	NLS	
Labeling in this paper	Human	v3	v2	v12	v1	
(98)	Bovine	CKI-α	-	CKI-αL	-	Identification of L insert
(99)	Human	-	CK1α	-	-	Identification of S insert; localization of *CSNK1A1* gene to chr13q13
(25)	Human	-	hCKIα2	-	-	Authors mention “hCKIαL (also called CKIα3)” without any reference to source or sequence
(100)	Pig, rat	α-CKI	-	αL-CKI	-	Colocalization with microtubules, ER, Golgi, and synaptic vesicles
(9)	Rat	CKIα	-	CKIαL	-	Authors suggest the existence of additional splice variants
(101)	Human	CKIα1	CKIα2		CKIα3	Unclear composition of CKIα3
(102)	Chicken	CKIα	CKIαS	CKIαL	CKIαLS	The first demonstration that all four splice variants exist in a single type of cell
(61)	Human	-	CKIαS	CKIαL	-	Authors suggest that mRNA of CKIαL is less stable than CKIαS
(10)	Chicken	CKIα	CKIαS	CKIαL	CKIαLS	Localization of variants
(60)	*Danio rerio*	CK1α	CK1αS	CK1αL	CK1αLS	Localization, inhibition, and thermostability of variants
(8)	Human	-	-	-	CK1αLS	hnRNP-C and H_2_O_2_
(36)	*Danio rerio*	CK1α	CK1αS	CK1αL	-	C-term autophosphorylation
(103)	Human	-	-	-	CK1αLS	Functional significance of CK1αLS in nucleus
(104)	Human	CK1αNI	CK1αS	CK1αL	CK1αLS	Authors describe effect of pyrvinium on activation of splice variants

Overview of literature references dealing with the topic of CK1α splice variants.

ER, endoplasmic reticulum; hnRNP-C, heterogeneous nuclear ribonucleoprotein C.

Our rescue assay showed that only two CK1α variants, v2 and v3, which contain the entire kinase domain (N-segment) and lack the L-segment responsible for nuclear import of CK1α are able to fully rescue and act as negative regulators of the Wnt/β-catenin pathway. It was tempting to speculate that the L-segment renders CK1α inactive in rescue assays because it causes its nuclear localization. This hypothesis was, however, excluded based on the phenotype of CK1α-v1/v12 (K160N), which showed cytoplasmic localization but still failed to fully rescue the phenotype. We therefore speculate that the L-segment *per se* affects the substrate specificity, as previously proposed (9, 60, 61) or that this insert modifies CK1α protein–protein interactions. Several variants, in particular CK1α-v1/v12, its cytoplasmic variant CK1α-v1/v12 (K160N), and CK1α-like could partially rescue, but their phenotype was clearly different from CK1α-v2/v3. Thus, the interpretation of the partial rescue remains unclear. This is especially true in light of the fact that CK1α-v2/v3, which rescued fully, were indistinguishable from CK1α-v1 and CK1α-like, which rescued partially, in their ability to phosphorylate Ser45 of β-catenin in kinase assays.

We have failed to assign a function of CK1α splice variants lacking the functional kinase domain despite we tested two hypotheses on their possible function. The first “complementation” hypothesis tested the theoretical possibility that two splice variants can combine and form a fully functional kinase. The second “regulatory” hypothesis tested the possibility that splice variants containing the C-terminus of CK1α can regulate the activity of the canonical CK1α variants (36). We were unable to experimentally support any of these two possibilities. However, CK1α plays a plethora of roles in cells (for review, see Fulcher and Sapkota (62); Jiang *et al.* (7)) and these truncated splice variants may have a role in other processes beyond the Wnt/β-catenin signaling pathway.

Our study included also the lesser-known CK1α-like, known also as CK1α2 (63) or MGC33182 (64). In stark contrast to the well-documented CK1α isoforms, CK1α-like remains poorly characterized, meriting its recent inclusion in the “dark kinome” database of understudied kinases (13). CSNK1A1L, the gene encoding CK1α-like, contains no introns and it is expressed as a single splice variant. CK1α-like shares a substantial 90.5% identity with CK1α-v2 throughout its protein sequence. However, our data provide clear evidence demonstrating that CK1α-like is not redundant with CK1α in the Wnt/β-catenin pathway: Deletion of CK1α-like, either alone or together with CK1α KO, does not show any obvious phenotype and CK1α-like failed to rescue the phenotype in CK1α KO. This is surprising as CK1α-like can still phosphorylate β-catenin on Ser45. Intriguingly, CK1α-like is absent in mice, but a closely related homolog of CK1α-like with 98% sequence identity in the catalytic domain is present in zebrafish (65). The biological role of CK1α-like thus remains elusive, with experimental evidence limited to the transcript level (Q8N752; The UniProt Consortium *et al.* (66)) and reports from few proteomic studies (see below). Several studies have tangentially associated CK1α-like with different malignancies. For instance, it has been linked to Wnt signaling–related genes in the context of methylation differences between patients with osteoporotic hip fractures and those with hip osteoarthritis (67). Furthermore, somatic mutations in the CSNK1A1L gene have been identified in colorectal cancer (68). A recent study has reported potential protein–protein interactions involving CK1α-like including interaction with β-arrestin (69). Mass spectrometry revealed also that CK1α-like, as well as CK1α, interact with the Hedgehog signaling pathway receptor Patched 1 (70). Furthermore, an analysis of protein interaction networks in the human liver detected potential interactions between CK1α-like and an enhancer binding protein, CEBPE, and junctional adhesion molecule JAM2, using the yeast two-hybrid system (71). Other possible interactions involving CK1α-like include associations with FAM170A, a nuclear transcription factor, which positively regulates the expression of heat shock genes (72, 73), the transcription factor JUN (74), and all three subunits of DNA replication and homologous recombination-associated replication protein A, RPA (75). Recently, two high-throughput studies which identified numerous additional interactors of CK1α-like have been published (76, 77). Despite these potential links to biological functions, none of these studies reported a functional analysis on the biological role of CK1α-like.

CK1 kinases are implicated in various diseases (for review, see Janovska *et al.* (78); Schittek and Sinnberg (79)), which has triggered development of (isoform-specific) small molecule inhibitors of CK1 and other CK1 modulators (for review, see Sunkari *et al.* (80)). As the field moves from the basic research to the development of therapeutics, we decided to set up an assay that allows to test the in-cell potency of the available CK1α inhibitors towards the CK1α/α-like variants. The response to inhibitors for some CK1α splice variants has been described previously (60). The report included four variants from *Danio rerio* and proposed that variants containing the L-segment have higher turnover, lower thermostability, and two times higher K_M_ for ATP, making them less sensitive to ATP-analog inhibitor, CKI-7. Our results do not support a role of the L-segment, because all tested CK1α variants (v1, v2, and v3) had similar affinity for the tested ligands. Interestingly, this was not the case for CK1α-like that was inhibited significantly less efficiently. Our NanoBRET assay represents an optimized version of CK1α NanoBRET published earlier (50), yielding results well in line with other functional cellular assays (50). We conclude that MU1742, given its excellent kinome-wide selectivity and high potency towards all tested CK1α variants in cellular environment, represents currently the best chemical probe to study CK1α biology.

In summary, we have produced and characterized a model system that allows to functionally test the role of CK1 variants in the Wnt/β-catenin pathway. The experimental system that we introduced helped us to uncover the molecular details of the negative regulation of Wnt/β-catenin pathway by CK1α. Furthermore, the tools generated are a perfect basis for the follow-up studies focused on other molecular and cellular functions of CK1α and CK1α-like.

## Experimental procedures

### Cell culture

T-REx-293 (Invitrogen, R71007) and all KO cell lines derived from them were cultured in the presence of high-glucose Dulbecco’s Modified Eagle Medium (DMEM, Gibco, 41966029) supplemented with 10% (v/v) fetal bovine serum (Gibco, 10270106) and 1% (v/v) penicillin/streptomycin (Biosera, XC-A4122/100), further on referred to as a complete DMEM. Cells were cultivated in the cell culture incubator at 37 °C under 95% (v/v) humidity and 5% (v/v) CO_2_. Cells were periodically tested for *mycoplasma* absence by PCR (FP- GGGAGCAAACAGGATTAGATACCCT, RP- TGCACCATCTGTCACTCTGTTAACCTC). When needed, monolayer of cells was rinsed by the PBS (140 mM NaCl; 8 mM Na_2_HPO_4_.12H_2_O; 1 mM KH_2_PO_4_; distilled H_2_O) and afterward 1× Trypsin/EDTA (Biosera, LM-T1706/100) was gently applied to the cells. After approximately 5 min of trypsinization in the incubator, 1× Trypsin/EDTA was neutralized by addition of the complete DMEM, and obtained suspension of cells was subsequently handled and cultured on different types of plates (TPP Techno Plastic Products AG) according to the experimental setup or maintenance.

### Transient transfection

Transient transfections were performed using PEI (Polysciences, 23966). Cells were plated a day in advance to be on the day of transfection at 50 to 60% confluency. Transfection mix was prepared according to the culture plate size. In case of one well of 24-well plate was mixture as follows. The total volume of serum/antibiotics-free DMEM was 50 μl. Half of the total volume of serum/antibiotics-free DMEM (25 μl) was incubated with 1.6 μl of PEI (1 μg/ml; pH 7.4) for 30 min, while the other half (25 μl) was mixed with DNA desired for transfection. Total amount of DNA was kept at 0.4 μg by the addition of pcDNA3 empty backbone (Invitrogen) when needed. Resulting DNA to PEI ratio was 1:4. Subsequently, both mixtures were merged and further incubated for 30 min. Each preparation step was accompanied by brief vortexing and centrifugation. Cells were treated by transfection mix and incubated for 6 h. After that time, medium was aspirated and substituted by the fresh complete DMEM, alternatively with treatment.

### Cloning and mutagenesis

Mammalian expression vectors of human CK1α variants and CK1α-like were constructed through Gateway cloning. Original entry clones pDONR223-CSNK1A1 (addgene, #23355) (81) and pDONR223-CSNK1A1L (addgene, #23784) (81) were adjusted using QuikChange II XL site-directed mutagenesis kit (Agilent, 200522) to repair the STOP codon. Entry clone pDONR223-CSNK1A1 was further edited using In-Fusion HD EcoDry Cloning Plus (Takara Bio, Inc; 638913) to reflect the sequences of all CK1α splice variants. All prepared entry clones were subsequently used for the construction of expression vectors with the destination vector pDEST-pcDNA3.1 N-term 3xFlag and Gateway LR Clonase II Enzyme mix (Invitrogen, 11791020).

Fragments of DNA containing the coding sequences of human CK1α-v1, CK1α-v2, CK1α-v3, CK1α-like, and mutant version CK1α-v2(K46R), respectively, were cloned into the plasmid pFastBac-His6-TEV (438-B) (addgene, #55219) for the purification of full-length kinases with an N-terminal His6 tag from insect cells. Fragment of DNA containing the coding sequence of human β-catenin was cloned into the plasmid pET-His6-GST-TEV (2G-T) (addgene, #29707) for the purification of the full-length β-catenin with an N-terminal His6 tag and an N-terminal GST tag from bacterial cells.

The sequences of all constructs in this study were verified by Sanger sequencing. Sequences of cloning and mutagenesis primers are available upon request.

### Immunocytofluorescence

T-REx-293 WT cells were seeded at low density on Corning Matrigel (VWR 734-1101)-coated coverslips in 24-well plates and transfected after 48 h of cultivation. Cells were transfected with 200 ng (Fig. 2) or 50 ng (Fig. 5) of expression vector per well and 200 ng (Fig. 2) or 350 ng (Fig. 5) of pcDNA3 empty backbone using PEI for 6 h. Afterward, fresh complete DMEM was applied on cells. Coverslips with cells were let growing for 20 h and subsequently were washed with PBS, fixed with 4% (v/v) paraformaldehyde, permeabilized with 0.1% (v/v) Triton X-100 (Sigma-Aldrich, T8787), washed with PBS, blocked with 1% (w/v) bovine serum albumin, and incubated with Flag M2 (Sigma-Aldrich, F1804) primary antibody overnight at 4 °C. The next day, cells were washed with PBS and incubated with Alexa Fluor 488 (Invitrogen, A21202) for 2 h. For the last 30 min, Alexa Fluor 568 Phalloidin (Invitrogen, A12380) was added. Cells were subsequently washed once with PBS, and 4′,6-diamidino-2-phenylindole (Invitrogen, D1306) was diluted in PBS and applied for 10 min as the second wash. Additional two PBS washes were performed. Coverslips were mounted with DAKO (Agilent, S3023) on microscopic slides and let dry overnight. Coverslips were scanned as multi-channel single planes as well as z-stacks using a Leica SP8 system.

Quantification of immunocytofluorescence images was performed on single plane images as well as z-stack images in which layers were processed using sum of pixel values along a single z-axis point through ZProjection – SumSlices in Fiji (ImageJ 1.53q) (82). Further, the areas of the whole cells and their nuclei were segmented from multi-channel tiff images using Cellpose (v0.7.1) (83) based on Alexa Fluor 568 Phalloidin (whole cell) and 4′,6-diamidino-2-phenylindole (nucleus) staining. Resulting segments (labels) were converted to regions of interest (ROIs) in Fiji software (https://fiji.sc/) using LabelsToROIs plugin (84). Resulting ROIs were used for the quantification of fluorescent signal of overexpressed CK1α splice variants and CK1α-like isoform. The signal was quantified as a mean pixel intensity in two areas, nuclear ROI and cytoplasm. The latter was quantified in ROI as a result of subtracting nuclear ROI from the whole cell ROI. Obtained mean pixel intensities were further processed to get nucleus/cytoplasm ratio. These ratios were visualized in superplots using SuperPlotsOfData web application (https://huygens.science.uva.nl/SuperPlotsOfData/) (85). Statistical analysis was performed on the means of individual replicates in GraphPad Prism 8. Particular statistical tests are described in each figure.

### CRISPR/Cas9 KO cell lines

Design of guide RNA (gRNA) sequences was carried out through the CRISPick-CRISPRko online designing tool available at https://portals.broadinstitute.org/gppx/crispick/public (86, 87). Two oligonucleotides for every gRNA were deduced, further on referred to as a top and a bottom oligo (CSNK1A1 top oligo: **CACCg**CATATCGGGCAGTGCCAGTG, bottom oligo: **AAAC**CACTGGCACTGCCCGATATG**c**; CSNK1A1L top oligo: **CACCg**CTGACCATACCAGTGCATGT, bottom oligo: **AAAC**ACATGCACTGGTATGGTCAG**c**). Top oligo contains exactly obtained gRNA sequence, and together with reverse complement bottom oligo both include additional oligonucleotides (in bold) for optimal mutual annealing and cloning into the one of vector backbones (for CSNK1A1: pSpCas9(BB)-2A-GFP, addgene #48138; for CSNK1A1L: pU6-(BbsI)_CBh-Cas9-T2A-mCherry, addgene #64324) (88, 89). Backbones expressed fluorescently tagged Cas9 nuclease and one gRNA. Cloning procedure was based on the Golden Gate assembly and was carried out according to the protocol described by Ran *et al.* (89). Prepared vectors were validated by Sanger sequencing using primer in the region of U6 promoter with the following sequence 5′-GAGGGCCTATTTCCCATGATTCC-3′.

Plasmids were transfected alone or in combination to achieve single as well as double KO lines. Lipofectamine 2000 (Invitrogen, 11668027) was used according to the manufacturer’s instructions to achieve higher transfection efficiency. After 24 h post transfection with CRISPR plasmid/s, cells underwent fluorescence-activated cell sorting. Singe cells were sorted on BD FACSAria Fusion into 96-well plates prefilled with 50 μl of complete DMEM supplemented by additional 10% (v/v) fetal bovine serum. The next day, additional 50 μl of regular complete DMEM were added to each well. Cells were further cultured and expanded till they achieved sufficient amount. Part of the cells of each monoclonal cell line was used for the isolation of genomic DNA with DirectPCR Lysis Reagent – Cell (Viagen Biotech, 302-C) and 0.5 mg/ml Proteinase K (Thermo Fisher Scientific, EO0491) according to the manufacturer’s instructions. Isolated genomic DNA was used for the PCR screening of CRISPR-targeted locus together with DreamTaq Polymerase (Thermo Fisher Scientific, EP0702) and designed screening primers (for CSNK1A1: FP-CCTTCAAATCCTCAGCCAAG, RP-GCACACTTGTTGCTTCTGCT; for CSNK1A1L: FP-ACGGCAGGCTGGTTCTATTA, RP-TTTGCCTCTCAGGATGACAA). Product obtained from the PCR of CRISPR-targeted locus further underwent simplified restriction fragment length polymorphism analysis as the successful targeting with selected gRNA should disrupt recognition site for the restriction enzyme (for CSNK1A1: BsrI; for CSNK1A1L: NspI) and indicate creation of a KO line. Reaction products were visualized site by site on a 2% (w/v) agarose gel (Serva, 11404) against ZipRuler Express DNA Ladder Set (Thermo Fisher Scientific, SM1373). DNA amplicons with CRISPR/Cas9-mediated modification exhibited one band (for CSNK1A1: 583 bp; for CSNK1A1L: 674 bp) while the ones without modification exhibited two bands (for CSNK1A1: 417 + 166 bp; for CSNK1A1L: 437 + 237 bp). Modified clones were further submitted for WB analysis and final confirmation was carried out through the next generation sequencing of the PCR product on MiSeq Illumina platform according to the previously described protocol (90). Analysis was performed in the Integrative Genomics Viewer software – IGV (https://igv.org/) (91).

### SDS-PAGE and Western blotting

Protein samples were prepared by lysis of cells with 2× Laemmli buffer (4% (w/v) SDS, 20% (v/v) glycerol, 0.125 M Tris–HCl at pH 6.8, 10% (v/v) β-mercaptoethanol, 0.004% (w/v) bromphenol blue), sonication, and following boiling of samples for 5 min at 95 °C. For the separation, different densities of polyacrylamide gels were used. PageRuler Plus Prestained Protein Ladder (Thermo Fisher Scientific, 26620) was used as a size standard. Electrophoresis was performed in the 1× Running buffer (190 mM glycine; 25 mM Tris; 0.1% (w/v) SDS) at 80 to 140 V until the desired resolution was achieved. Western blot transfer to the methanol-activated polyvinylidene difluoride membranes (Immobilon-P PVDF, Millipore, IPVH00010) was carried out in the 1× Transfer buffer (190 mM glycine; 25 mM Tris; 20% (v/v) methanol) at 105 V for 75 min for 8% gels and +5 V for every additional 2% of gel density. Membranes were afterward blocked in the 5% (w/v) skimmed milk in TBST buffer (100 mM NaCl; 10 mM Tris–HCl at pH 7.6; 0.08% (v/v) Tween20) for 1 h at a room temperature. Primary antibodies (order of appearance: CK1α – abcam ab206652, 1:1000; β-actin – Cell Signaling Technology cs-4970, 1:2000; LRP6 total – Cell Signaling Technology cs-2560, 1:1000; LRP6 pS1490 – Cell Signaling Technology cs-2568, 1:500; DVL3 – Cell Signaling Technology cs-3218, 1:1000; inactive β-catenin – Cell Signaling Technology cs-9565, 1:500; total β-catenin – BD 610153, 1:2000; Flag M2 – Sigma-Aldrich F1804, 1:1000; α-tubulin – Sigma-Aldrich T6199, 1:2000; CK1α-like – abcam ab180490, 1:1000; β-catenin pS45 – Cell Signaling Technology cs-9564, 1:500) were diluted in 5% (w/v) skimmed milk in TBST buffer and incubated with membranes overnight at 4 °C. The next day, membranes were washed three times for 15 min in TBST buffer and then incubated with the peroxidase-conjugated secondary antibodies (Anti-Rabbit IgG, Sigma-Aldrich A6667; Anti-Mouse IgG, Sigma-Aldrich A6782) diluted 1:5000 in 5% (w/v) skimmed milk in TBST buffer for 1 h at room temperature. After three 15 min washes in TBST buffer, membranes were covered with chemiluminescent HRP substrate according to the manufacturer’s instructions (Millipore, WBKLS0500), and emitted light was captured in a camera chamber Fusion SL Vilber Lourmat using provided software.

ImageJ software (https://imagej.net/ij/) (92) was used for the postcapture processing of images in terms of their cropping and linear adjustments of brightness and contrast. Furthermore, the software was used for the quantification of obtained signals. The built-in function for gel analysis was utilized. Specifically, the gated areas under the curves of plotted lanes of interest, which correspond to the protein signals of interest, were measured. These measurements were subsequently normalized to a loading control.

### Dual luciferase assay

Dual luciferase method of assaying the canonical Wnt signaling pathway is also known as TopFlash/Renilla assay or TopFlash for short. It uses Firefly and Renilla luciferases for relative quantification of TCF/LEF-driven genes expression, which are dependent on the Wnt/β-catenin pathway activation (29).

Cells in each well of 24-well plate were cotransfected with several vectors using PEI transfection (see Transient transfection). First of the vectors was 100 ng of Super 8× TopFlash (93), which encodes Firefly luciferase under the control of eight TCF/LEF-binding sites. This way, the activation of the canonical Wnt/β-catenin signaling pathway results not only in the expression of its target endogenous genes but also in the expression of the Firefly luciferase. As an internal control, 100 ng of pRL-TK vector (Promega), expressing Renilla luciferase under the constitutive promoter, was cotransfected. Thus, Renilla luciferase signal reflects the total gene expression in the sample. Cells were cotransfected with 50 ng of the vector expressing cDNA of the particular CK1α splice variant or CK1α-like (see Cloning and mutagenesis). Total amount of transfected DNA per well was buffered to 400 ng by addition of the pcDNA3 (Invitrogen) empty backbone to transfection mix. Cells were transfected for 6 h and subsequently treated.

To inhibit autocrine secretion of Wnt lipoglycoproteins, the cells were treated with 0.5 μM porcupine inhibitor LGK974 (Stem RD, L974-010) for 16 h. To stimulate Wnt signaling cascade, either Wnt-3a conditioned medium or Wnt-3a recombinant protein (R&D Systems, 1324-WN) at a concentration of 80 ng/ml was used for 16 h. To potentiate the stimulation, recombinant protein R-spondin-1 (RSPO1, PeproTech, 120-38) was used at a concentration of 25 ng/ml for 16 h. Control stimulations were carried out with either a control conditioned medium or 0.1% (w/v) bovine serum albumin (Serva, 11930.03) in PBS, which was used as a solvent of recombinant proteins.

Dual luciferase assay was carried out by the commercially available Dual-Luciferase Reporter Assay System (Promega, E1960), according to the manufacturer’s instructions. Briefly, the next day after transfection, cells were washed once with PBS, all liquid was aspirated, and cells were dry frozen at −80 °C for 1 h. After the plate reached room temperature, passive lysis buffer from the kit was applied on the cells. Lysis lasted for 20 min at room temperature while rocking the plate. Then, lysate was moved to flat bottom strips (Thermo Fisher Scientific, 7566). Luciferase Assay Reagent II was added to lysates in the strips, and immediately, luminescence produced by Firefly luciferase was measured by a Hidex Bioscan Plate Chameleon Luminometer. In the next step, Stop&Go reagent was added to the wells. This resulted on one hand in the quenching of the signal produced by Firefly luciferase, and on the other hand, it provided the substrate for Renilla luciferase, in which luminescence was immediately measured.

Obtained raw luminescence units for the TopFlash luciferase were divided/normalized by raw luminescence units for Renilla luciferase. Resulting value represent relative luminescence units corresponding to the relative activity of the Wnt/β-catenin signaling pathway. Each value of a given replicate was further normalized to the mean value of the whole replicate. Eventually, each value across replicates was normalized by the mean value of controls (T-REx-293 WT Ctrl/pcDNA/-WNT3A, -RSPO1) across replicates. Data normalized in this way were plotted and statistically evaluated in GraphPad Prism 8 software (https://www.graphpad.com/) using Two-Way or Three-Way ANOVA with false discovery rate correction for multiple comparisons (Two-stage step-up method of Benjamini, Krieger, and Yekutieli at Q = 0.05).

### Protein expression and purification

#### Protein expression in insect cells

To generate viruses enabling the production of proteins in insect cells, recombinant plasmids of CK1α-v1, CK1α-v2, CK1α-v3, CK1α-like, and CK1α-v2(K46R), respectively, were transposed into bacmids using the Tn7 transposition method in *Escherichia coli* DH10Bac cells. Viral particles were obtained by transfection of the corresponding recombinant bacmids into the *Sf*9 cells using the Insect GeneJuice Transfection Reagent and further amplification. Proteins were expressed in 300 ml of High Five (Hi5) insect cells (infected at 1.5 × 10^6^ cells/ml) with the corresponding P1 virus at multiplicity of infection > 1. Cells were harvested 48 h postinfection, washed with 1× PBS, and stored at −80 °C until further use.

#### Protein expression in bacterial cells

β-catenin was expressed in *E. coli* BL21 (DE3)-RIPL bacterial cells. Cells were grown at 37 °C to optical density 0.5 at 600 nm. Expression of His6-GST-tagged protein was induced by 0.5 mM IPTG, followed by an overnight expression at 16 °C. Cells were then harvested, washed with 1× PBS, and stored at −80 °C until purification.

### Protein purification

#### CK1α variants from insect cells

The thawed insect cells, expressing CK1α-v1, CK1α-v2, CK1α-v3, CK1α-like, and CK1α-v2(K46R), respectively, were resuspended in lysis buffer (50 mM Tris–HCl at pH 8.0; 500 mM NaCl; 10% (v/v) glycerol; 0.4% (v/v) Triton-X; 10 mM imidazole; 1 mM DTT) containing protease inhibitors (1 μg/ml pepstatin; 7.5 μg/ml benzamidine; 7.1 μg/ml leupeptin; 3 μg/ml aprotinin) and 25 U of benzonase per ml of lysate. The cleared lysate was passed through 1.5 ml of Ni-NTA beads (Qiagen), equilibrated with buffer containing 50 mM Tris–HCl at pH 8.0, 500 mM NaCl, 10 mM imidazole, and 1 mM DTT. The kinases were eluted with an elution buffer containing 50 mM Tris–HCl at pH 8.0, 500 mM NaCl, 500 mM imidazole, and 1 mM DTT. The elution fractions were loaded onto a Superdex 75 column (Cytiva) and eluted with 25 mM Tris–HCl at pH 7.5, 250 mM NaCl, and 1 mM DTT. Fractions containing homogeneous CK1α-v1, CK1α-v2, CK1α-v3, CK1α-like, and CK1α-v2(K46R), respectively, were concentrated, and glycerol was added to a final concentration of 10% (v/v) before they were snap-frozen in liquid nitrogen and stored at −80 °C.

#### β-catenin from *E. coli*

The thawed bacterial cells expressing β-catenin were resuspended in lysis buffer (50 mM Tris–HCl at pH 8.0; 500 mM NaCl; 10 mM imidazole; 1 mM DTT) containing protease inhibitors (1 μg/ml pepstatin; 7.5 μg/ml benzamidine; 7.1 μg/ml leupeptin; 3 μg/ml aprotinin) and 10 mg of lysozyme per ml of lysate. The cleared lysate was passed through 2 ml of Ni-NTA beads (Qiagen), equilibrated with a buffer containing 50 mM Tris–HCl at pH 8.0, 500 mM NaCl, 10 mM imidazole, and 1 mM DTT. β-catenin was eluted with an elution buffer containing 50 mM Tris–HCl at pH 8.0, 500 mM NaCl, 500 mM imidazole, and 1 mM DTT. The elution fractions were loaded onto a Superdex 200 column (Cytiva) and eluted with 25 mM Tris–HCl at pH 7.5, 250 mM NaCl, and 1 mM DTT. Fractions containing homogeneous β-catenin were concentrated, and glycerol was added to a final concentration of 10% (v/v) before they were snap-frozen in liquid nitrogen and stored at −80 °C.

### *In vitro* kinase assay

#### IVKA: Autophosphorylation assay

Autophosphorylation assay was performed using 1 μg of 6xHis-CK1α-like, 6xHis-CK1α-v2(K46R) kinase dead mutant as a control and 6xHis-CK1α variants v2, v3, v1, respectively. Reactions were carried out at room temperature and two time points (1 h, overnight) in the presence of 1 mM ATP and overnight in the absence of ATP as a control. Reactions were set in the buffer containing 25 mM Tris–HCl at pH 7.5, 250 mM NaCl, 1 mM DTT, 10 mM MgCl_2_, and 1mM EDTA. Reactions were stopped by the addition of 2× Laemmli buffer and boiling for 1 min at 95 °C. Reactions underwent SDS-PAGE on 12% (v/v) acrylamide gel which was subsequently stained with Coomassie Blue R-250 solution (0.25% (w/v) Brilliant Blue R (Sigma-Aldrich, B0149)) in H_2_O:methanol:glacial acetic acid (6:3:1, v/v) for 1 h. Gel was destained in the same solution without Brilliant Blue R for 1 to 2 h to overnight. Photo of gel was captured in a camera chamber Fusion Solo S Vilber Lourmat using provided software. ImageJ software (92) was used for the postcapture processing of images.

#### IVKA: β-catenin as a substrate

*In vitro* kinase assay (IVKA) with 6xHis-CK1α splice variants (v2, v2(K46R), v3, v1), as well as 6xHis-CK1α-like and 6xHis-GST-FL-β-catenin as a substrate were carried out in the buffer containing 25 mM Tris–HCl at pH 7.5, 250 mM NaCl, 1 mM DTT, 10 mM MgCl_2_, 1mM EDTA, and 1 mM ATP. Molar ratio was set to 1:10, 0.35 μM kinase and 3.5 μM substrate in 25 μl reaction mix. Reaction with just substrate without kinase (−) as well as 6xHis-CK1α-v2(K46R) kinase dead mutant served as controls. Reaction time was 1 h at room temperature, and reactions were stopped by the addition of 2× Laemmli buffer and boiling for 1 min at 95 °C. Each reaction was divided and loaded into several SDS-PAGE gels (various acrylamide percentages) which underwent either WB or Coomassie Blue R-250 staining to provide a loading control.

### NanoBRET target engagement assay

CK1α isoforms and mutants were cloned in frame with an N-terminal NanoLuc-fusion. The plasmid was transfected into HEK293T cells using FuGENE 4K (Promega, E5911) and proteins were allowed to express for 20 h. Serially diluted inhibitor and 1 μM NanoBRET tracer K10 (Promega, N2642) were pipetted into low volume white 384-well plates (Greiner, 784075) using an Echo acoustic dispenser (Labcyte). The corresponding protein-transfected cells were added and reseeded at a density of 2 × 10^5^ cells/ml after trypsinization and resuspending in Opti-MEM without phenol red (Life Technologies). The system was allowed to equilibrate for 2 h at 37 °C/5% (v/v) CO_2_ prior to BRET measurements. To measure BRET, NanoBRET NanoGlo Substrate was added as per the manufacturer’s protocol, and filtered luminescence was measured on a PHERAstar FSX plate reader (BMG Labtech) equipped with a luminescence filter pair (450 nm BP filter (donor) and 610 nm LP filter (acceptor)). For measurement of permeabilized cells, 50 μg/ml digitonin was added after readout, followed by an additional readout. Competitive displacement data were then graphed using GraphPad Prism 9 software using a normalized 3-parameter curve fit with the following equation: Y = 100/(1 + 10ˆ(X-LogIC_50_)).

## Data availability

The data supporting the findings of this article are available from the corresponding author.

## Supporting information

This article contains supporting information (21, 22, 94, 95, 96, 97).

## Conflict of interest

B.-T. B. is the CEO and a shareholder of CELLinib GmbH, Frankfurt, Germany. All other authors declare that they have no conflicts of interests with the contents of this article.
