# Supplementary material for: Splice variants of CK1α and CK1α-like: Comparative analysis of subcellular localization, kinase activity, and function in the Wnt signaling pathway
Source: J Biol Chem. 2024 May 23;300(7):107407. doi: 10.1016/j.jbc.2024.107407 (PMC11255964; doi:10.1016/j.jbc.2024.107407)

SUPPORTING INFORMATION

Running title: **Splice variants of CK1α and CK1α-like in the Wnt signaling**

Title: **Splice variants of CK1α and CK1α-like: Comparative analysis of subcellular localization, kinase activity and function in the Wnt signaling pathway**

Tomáš Gybeľ[1], Štěpán Čada[1], Darja Klementová[1,2], Martin P. Schwalm[3,4,5], Benedict-Tilman Berger[3,4], Marek Šebesta[2], Stefan Knapp[3,4,5], Vítězslav Bryja[1,6]*

1) Department of Experimental Biology, Faculty of Science, Masaryk University, Brno, 62500, Czech Republic

2) CEITEC-Central European Institute of Technology, Masaryk University, 625 00 Brno, Czech Republic

3) Institute for Pharmaceutical Chemistry, Johann Wolfgang Goethe-University, Max-von-Laue-Strasse 9, Frankfurt am Main, Germany

4) Structural Genomics Consortium, Johann Wolfgang Goethe-University, Max-von-Laue-Strasse 15, Frankfurt am Main, Germany

5) German Cancer Consortium (DKTK)/German Cancer Research Center (DKFZ), DKTK site Frankfurt-Mainz, 69120 Heidelberg, Germany

6) Department of Cytokinetics, Institute of Biophysics of the Czech Academy of Sciences, 612 65 Brno, Czech Republic

* Correspondence to:

prof. Mgr. Vítězslav Bryja, Ph.D.

Department of Experimental Biology

Faculty of Science, Masaryk University

Kamenice 5, D36/112

625 00 Brno

Czech Republic

tel.: +420 549 493 291

e-mail: bryja@sci.muni.cz

TABLE OF CONTENT

Figure S1
Figure S2
Figure S3
Figure S4
Figure S5
Uncropped Western blot (WB) membranes

Figure S1

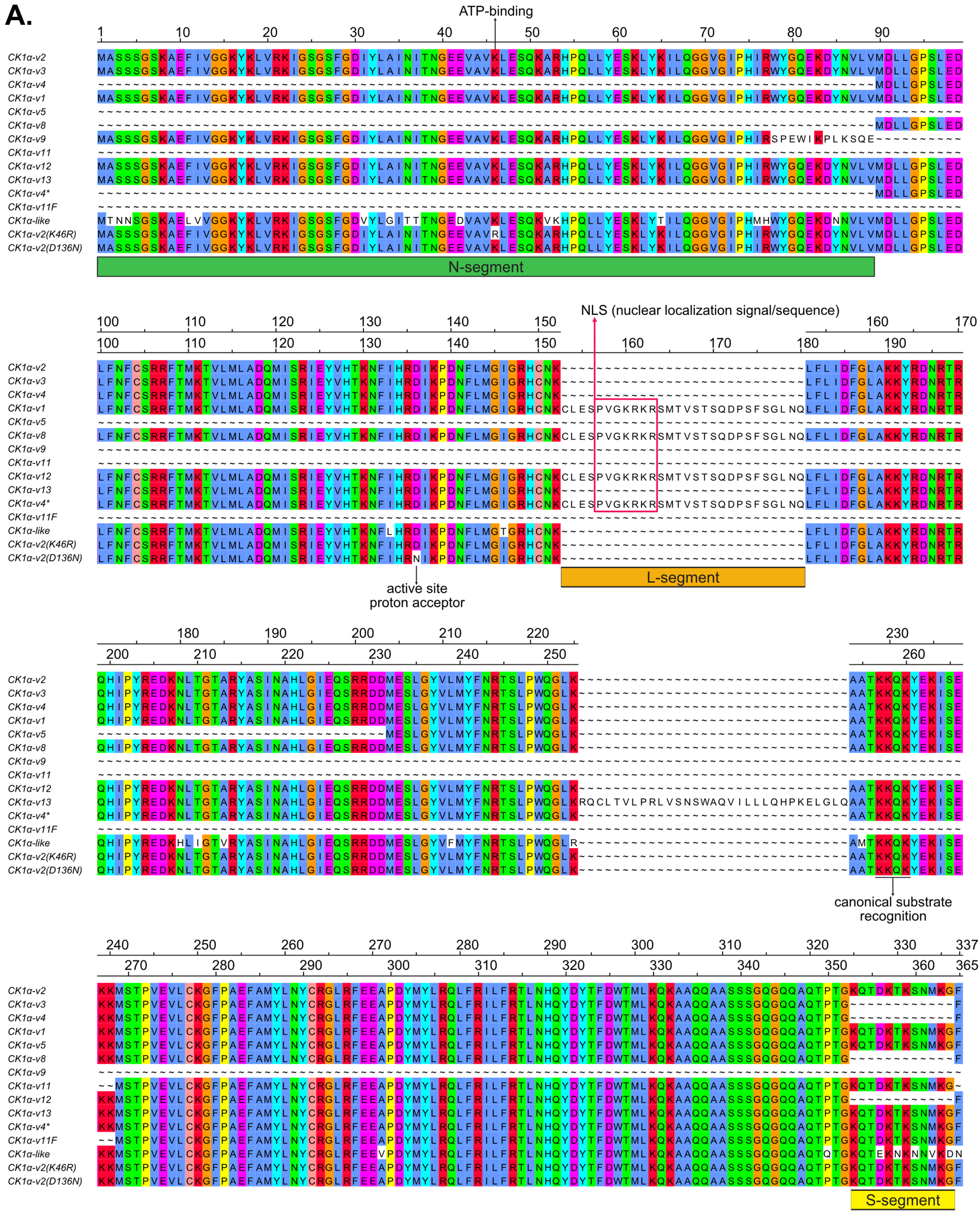

Figure S1

A - Multiple protein sequence alignment of CK1α splice variants, their mutants and CK1α-like. Under the alignment three segments are highlighted: green color represents N-terminal segment, orange color represents L-segment and yellow color represents S-segment. Highlighted are also important regulatory sites as ATP-binding site, active site, canonical substrate recognition site and nuclear localization signal (NLS). Alignment was created in Jalview 2.11.2.7 and modified Clustal color scheme was applied with identity threshold set to 45 % (94). Above the alignment ruler reflecting protein length is visualized: the top one is for CK1α-v2 and the bottom one is for CK1α-v1.

Figure S1

B.

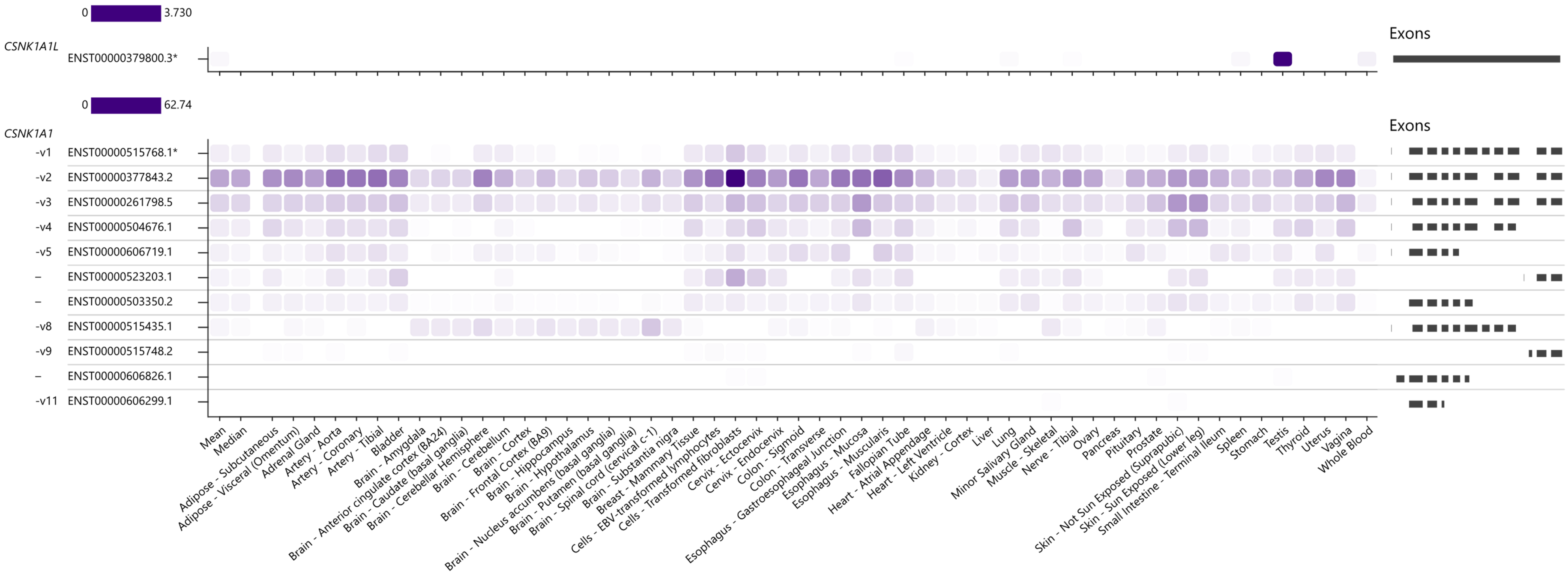

Figure S1

B - Visualization of CK1α splice variants and CK1α-like expression in various tissues. Taken and modified from the gnomAD v2.1.1 portal (last update 25. 8. 2023) (21, 22). Originally based on GTEx v7 RNA-Seq data. Color tiles reflect the level of expression (TPM-transcripts per million) in individual tissues as well as mean and median values across tissues (see the first two columns). Ensembl transcript IDs are based on the GRCh37/hg19 human genome assembly. *Transcript is the Ensembl canonical transcript for this gene.

Figure S1

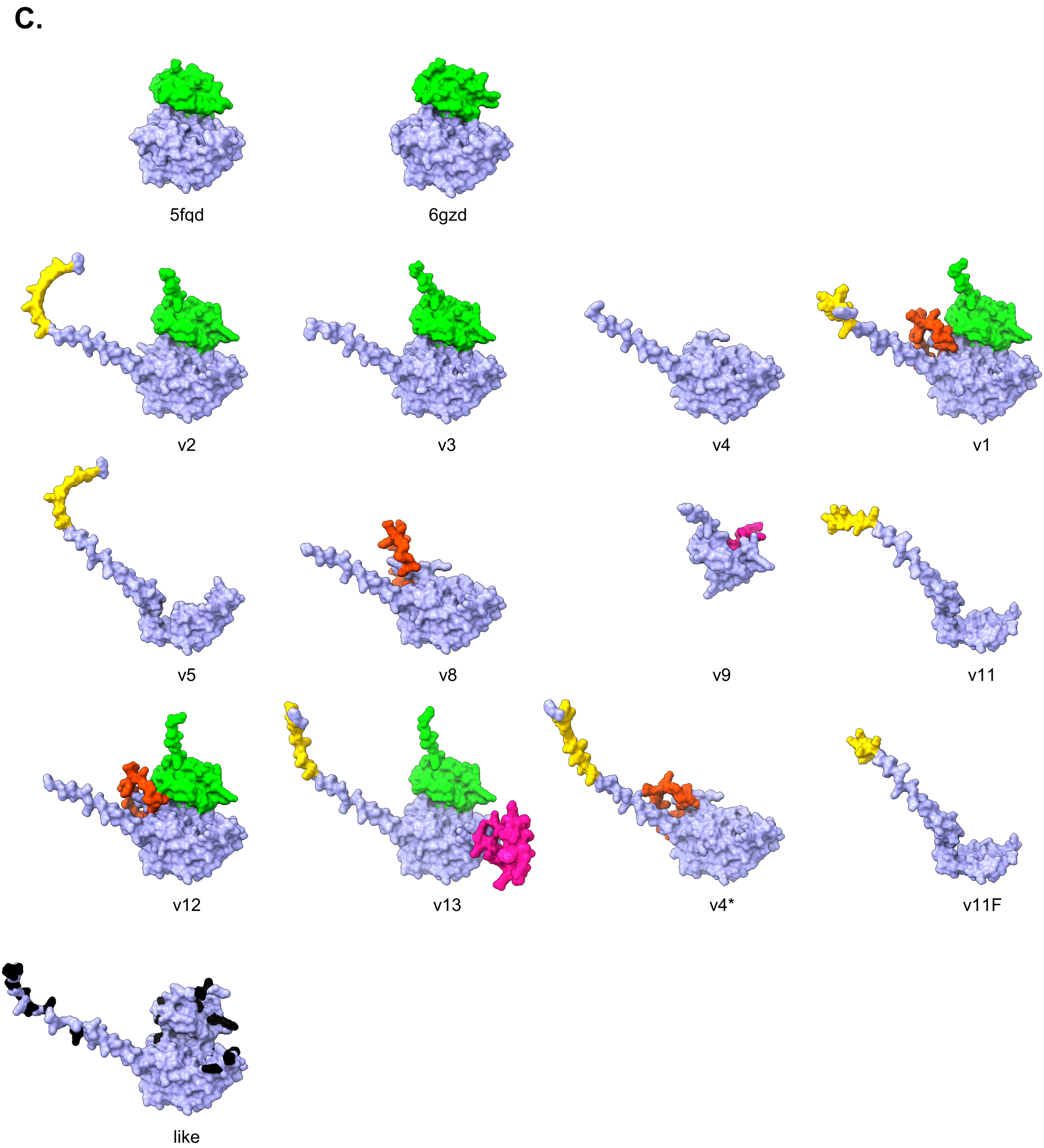

Figure S1

C – Crystal structures of CK1α (5fqd, 6gzd) and AlphaFold2 predictions of CK1α splice variants. N,L,S-segments are highlighted in color code from the Figure 1A. Predictions v2-AF_P48729, v4-AF_D6REM4, v8-AF_E7ETM0, v9-AF_D6RGE5, v12-AF_A0A5F9W493, v13-AF_U3KPX3 as well as prediction of CK1α-like structure - AF_Q8N752 were downloaded from AlphaFold Protein Structure Database (https://alphafold.ebi.ac.uk/) (95, 96). Predictions for v3, v1, v5, v11, v4* and v11F were created by ColabFold (97). Prediction of CK1α-like structure (AF_Q8N752) contains partial view of 32 aa residues (in black) which differ from CK1a-v2. Structures were superimposed but visualized separately.

Figure S2

A.

Gene	Protein	gRNA + **PAM**, Restriction site	PCR	RE	RFLP
CSNK1A1	CK1α	CATATCGGGCAGTGCCAGTG**AGG**	583bp	BsrI	417bp + 166bp
CSNK1A1L	CK1α-like	CTGACCATACCAGTGCATGT**GGG**	674bp	NspI	437bp + 237bp

B.

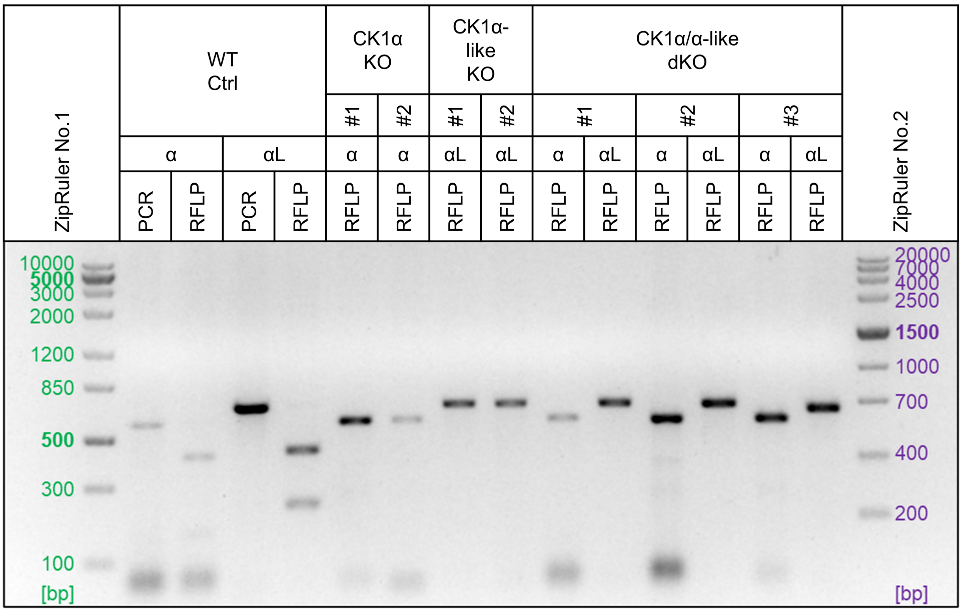

C.

Next Generation Sequencing Results				
CSNK1A1 gene - hg19: chr5 (+) 148892662 – 148892694				
WT		α	gctagCATATCGGGCAGTGCCA-GTGAGGttttt	
CSNK1A1L gene - hg19: chr13 (+) 37679146 – 37679188				
WT		αL	ttttcCTGACCATACCAGTGCA--TGTGGGggatgccaaccccac	
KO lines				Type of modification
CK1α KO	#1 (1B10)	α	gctagCATATCGGGCAGTGCCAAGTGAGGttttt	1nt ins
	#2 (2E10)	α	gctagCATATCGGGCAGTGCCAAGTGAGGttttt	1nt ins
CK1α-like KO	#1 (aL1)	αL	ttttcCTGACCATACCAGTGCAA-TGTGGGggatgccaaccccac<br>ttttcCTGACCATACCAGTGCA--TGTGGGggatgccaaccccac	1nt ins<br>2nt del
	#2 (aL2)	αL	ttttcCTGACCATACCAGTGCA--TGTGGGggatgccaaccccac<br>ttttcCTGACCATACCAGTGCAA-TGTGGGggatgccaaccccac	1nt del<br>1nt ins
CK1α/α-like dKO	#1 (4^2)	α	gctagCATATCGGGCAGTGCCAAGTGAGGttttt<br>gctagCATATCGGGCAGTGCCA-GTGAGGttttt	1nt ins<br>2nt del
		αL	ttttcCTGACCATACCAGTGCAAATGTGGGggatgccaaccccac	2nt ins
	#2 (5^2)	α	gctagCATATCGGGCAGTGCCAAGTGAGGttttt	1nt ins
		αL	ttttcCTGACCATACCAGTGCAA-TGTGGGggatgccaaccccac	1nt ins
	#3 (8^2)	α	gctagCATATCGGGCAGTGCCAAGTGAGGttttt	1nt ins
		αL	ttttcCTGACCATACCAGTGCA--TGTGGGggatgccaaccccac	29nt del

Figure S2

A - gRNA sequences used to produce single and compound KO lines of CK1α/α-like KO panel (Figure 3B). gRNAs were designed in line with the possibility of PCR screening (RFLP analysis). PAM is highlighted in bold and is not part of the gRNA sequence. Restriction/recognition site of the stated restriction enzyme used for PCR screening is underlined. Expected PCR product size and its restriction pattern is provided.

B - Agarose gel (2 %) showing RFLP analysis of KO clones.

C - NGS results confirming the status of KOs. PAM is displayed in blue and purple for individual CK1 isoforms. gRNA sequence is in black capitals. More sequences within one isoform represent different modifications of alleles. Dashes are involved for better vertical alignment of sequences. αL = α-like; dKO = double knockout; ins = insertion; del = deletion

Figure S3

A.

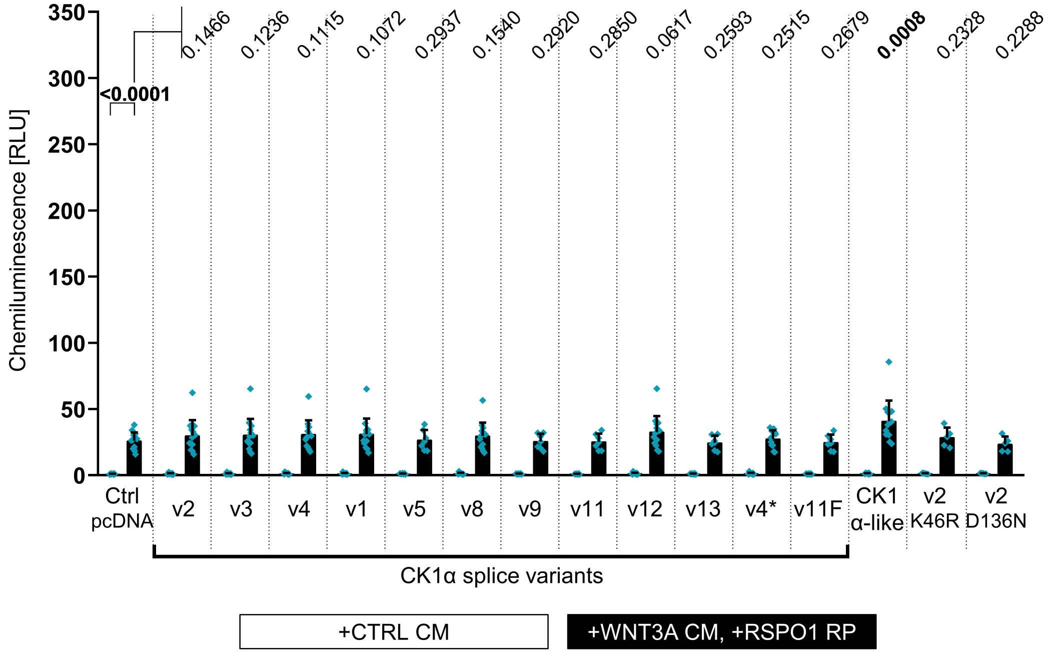

Figure S3

A - TopFlash assay - graph highlights the conditions of rescue experiment where T-REx-293 WT cells (blue data points) were transfected with expression vectors for individual CK1α splice variants, CK1α-like isoform or kinase dead mutants (K46R and D136N) of canonical variant CK1α-v2. The amount of transfected vector expressing splice variant was 50 ng. pcDNA condition served as the control of transfection. All conditions were pre-treated with porcupine inhibitor LGK974 (0.5 μM) to set basal state. Cells were further stimulated with Wnt-3a conditioned medium (WNT3A CM) with addition of R-spondin-1 recombinant protein (RSPO1 RP) (25 ng/ml) for 16 hours. The control conditions were treated with control conditioned medium (CTRL CM). Normalized ratios between TopFlash luciferase and Renilla luciferase are plotted. RLU - relative luminescence units. Statistical analysis: Outliers were identified through ROUT method (Q = 1 %) and excluded (4/684 measurements). Cleaned data were analyzed through Three-Way ANOVA with FDR correction for multiple comparisons (Two-stage step-up method of Benjamini, Krieger and Yekutieli; Q = 0.05; 2016 comparisons; q value is shown). Mean and S.D. error bars. n = 5-14 independent biological replicates.

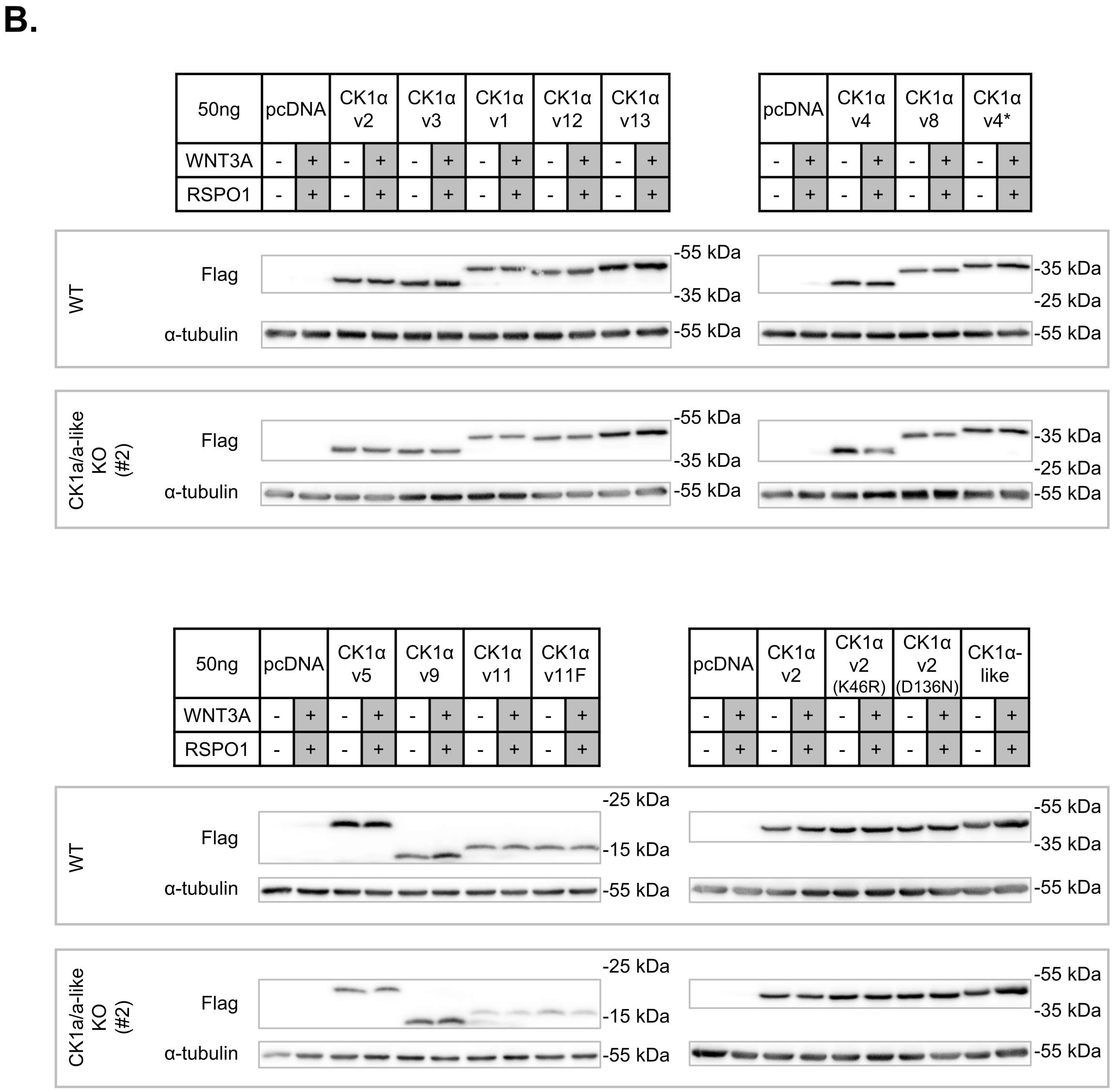

Figure S3

B - Western blot analysis of TopFlash lysates from Figure 4. Membranes were probed for Flag-tag to visualize transfected isoform. Probing for α-tubulin served as a loading control.

Figure S3

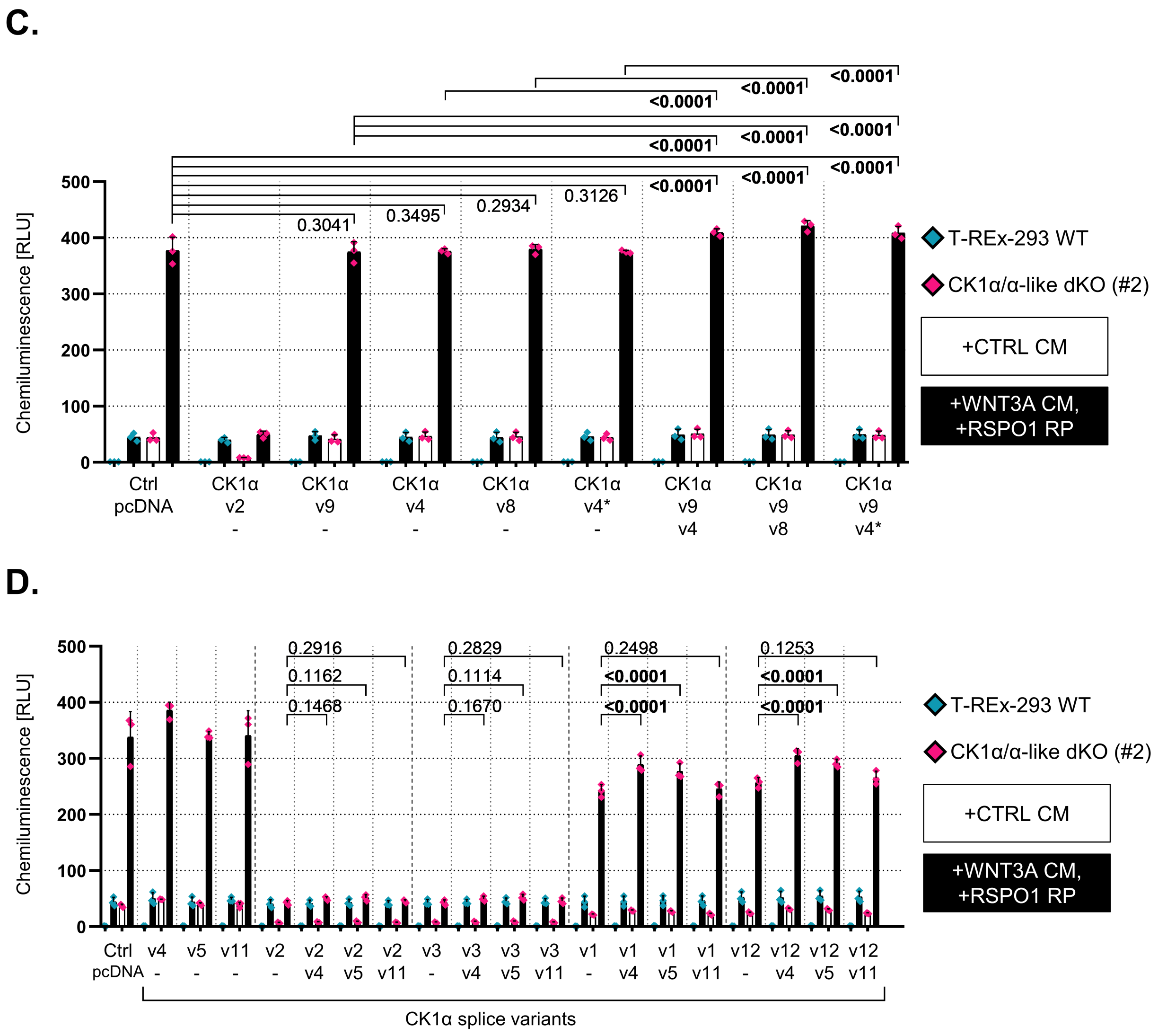

Figure S3

C - TopFlash assay - "complementary assay" with CK1α splice variants which combinations could potentially lead to the formation of active kinase. Empty pcDNA vector served as a control of transfection. The assay was performed on WT cells (blue data points) as well as CK1α/α-like KO cells (#2) (pink data points). The amount of each transfected vector was 50 ng. Cells were stimulated with Wnt-3a conditioned medium (WNT3A CM) with addition of R-spondin-1 recombinant protein (RSPO1 RP) (25 ng/ml) for 16 hours. The control conditions were treated with control conditioned medium (CTRL CM). All conditions were pre-treated with porcupine inhibitor LGK974 (0.5 μM) to set basal state. Normalized ratios between TopFlash luciferase and Renilla luciferase are plotted. RLU - relative luminescence units. Statistical analysis: Three-Way ANOVA with FDR correction for multiple comparisons (Two-stage step-up method of Benjamini, Krieger and Yekutieli; Q = 0.05; 630 comparisons; q value is shown). Mean and S.D. error bars. n = 3 independent biological replicates.

D - TopFlash assay - "phosphorylation decoy assay" with CK1α splice variants in combinations potentially leading to mutual inhibition. Empty pcDNA vector served as a control of transfection. The assay was performed on WT cells (blue data points) as well as CK1α/α-like KO cells (#2) (pink data points). The amount of each transfected vector was 50 ng. Cells were stimulated with Wnt-3a conditioned medium (WNT3A CM) with addition of R-spondin-1 recombinant protein (RSPO1 RP) (25 ng/ml) for 16 hours. The control conditions were treated with control conditioned medium (CTRL CM). All conditions were pre-treated with porcupine inhibitor LGK974 (0.5 μM) to set basal state. Normalized ratios between TopFlash luciferase and Renilla luciferase are plotted. RLU - relative luminescence units. Statistical analysis: Three-Way ANOVA with FDR correction for multiple comparisons (Two-stage step-up method of Benjamini, Krieger and Yekutieli; Q = 0.05; 3160 comparisons; q value is shown). Mean and S.D. error bars. n = 3 independent biological replicates.

Figure S4

A.

Gene	Protein	gRNA + **PAM**
GSK3A	GSK3α	CTCATTTGGGGTCGTGTACC**AGG**
GSK3B	GSK3β	GATCTAGCTTTCTCATGATC**TGG**

B.

Next Generation Sequencing Results				
GSK3A gene - hg19: chr19 (-) 42744167 – 42744199				
WT		GSK3α	aatggCTCATTTGGGGTCGTGT-ACCAGGcacgg	
GSK3B gene - hg19: chr3 (+) 119666160 – 119666192				
WT		GSK3β	acagtGATCTAGCTTTCTCATGATCTGGagctc	
CSNK1A1 gene - hg19: chr5 (+) 148892662 – 148892694				
WT		α	gctagCATATCGGGCAGTGCC--AGTGAGGttttt	
KO lines				Type of modification
GSK3α/β dKO	#3 (D5)	GSK3α	aatggCTCATTTGGGGTCGTGTTACCAGGcacgg	1nt ins
		GSK3β	acagtGATCTAGCTTTCTCATGATCTGGagctc	8nt del
CK1α/α-like + GSK3α/β tetraKO	#1 (3)	α/αL	based on CK1α/α-like dKO (#2)	
		GSK3α	aatggCTCATTTGGGGTCGTGTTACCAGGcacgg	1nt ins
		GSK3β	acagtGATCTAGCTTTCTCATGATCTGGagctc	4nt del
	#2 (12)	α/αL	based on CK1α/α-like dKO (#2)	
		GSK3α	aatggCTCATTTGGGGTCGTGTTACCAGGcacgg	1nt ins
		GSK3β	acagtGATCTAGCTTTCTCATGATCTGGagctc<br>acagtGATCTAGCTTTCTCATGATCTGGagctc	4nt del<br>4nt del
	#3 (13)	α/αL	based on CK1α/α-like dKO (#2)	
		GSK3α	aatggCTCATTTGGGGTCGTGTTACCAGGcacgg<br>aatggCTCATTTGGGGTCGTGTGACCAGGcacgg	1nt ins<br>1nt ins
		GSK3β	acagtGATCTAGCTTTCTCATGATCTGGagctc	2nt del
GSK3α/β + CK1α tripleKO	#1 (4)	GSK3α/β	based on GSK3α/β dKO (#3)	
		CK1α	gctagCATATCGGGCAGTGCCA-AGTGAGGttttt<br>gctagCATATCGGGCAGTGCCATAGTGAGGttttt	1nt ins<br>2nt ins
	#2 (7)	GSK3α/β	based on GSK3α/β dKO (#3)	
		CK1α	gctagCATATCGGGCAGTGCCA-AGTGAGGttttt	1nt ins
	#3 (8)	GSK3α/β	based on GSK3α/β dKO (#3)	
		CK1α	gctagCATATCGGGCAGTGCCA-AGTGAGGttttt<br>gctagCATATCGGGCAGTGCC--AGTGAGGttttt	1nt ins<br>1nt del

Figure S4
A - gRNA sequences used to produce single and compound KO lines of GSK3α/β KO panel (Figure 7B). PAM is highlighted in bold and is not part of the gRNA sequence.
B - NGS results confirming the status of KOs. PAM is displayed in color code corresponding to individual targeted genes (see WT forms). gRNA sequence is in black capitals. More sequences within one isoform represent different modifications of alleles. Dashes are involved for better vertical alignment of sequences. α = CK1α; αL = CK1α-like; dKO = double knockout; ins = insertion; del = deletion

C.

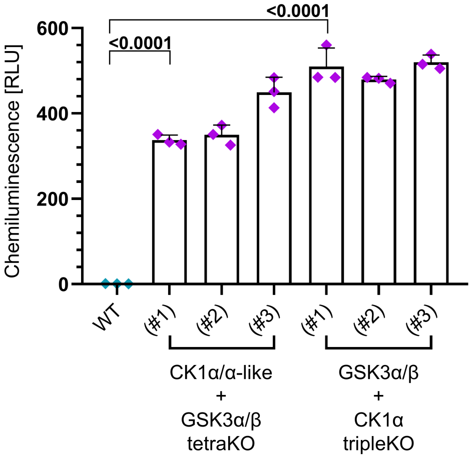

D.

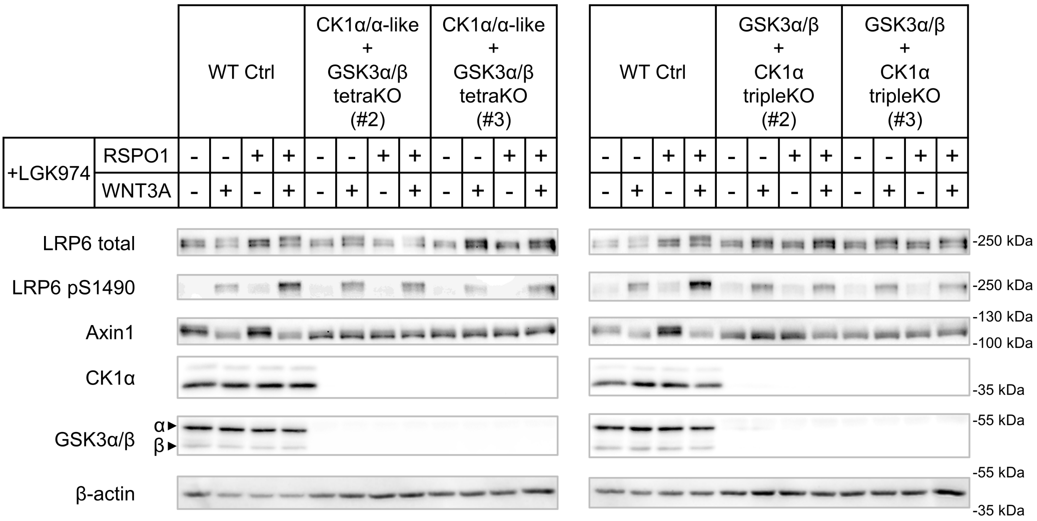

Figure S4

C - TopFlash analysis of CK1α/α-like + GSK3α/β tetraKO and GSK3α/β + CK1α tripleKO clones showing basal state of canonical Wnt signaling. Cells were treated for 16 hours with control conditioned medium (CTRL CM) and LGK974 (0.5 μM). Normalized ratios between TopFlash luciferase and Renilla luciferase are plotted. RLU - relative luminescence units. Statistical analysis: Ordinary one-way ANOVA with FDR correction for multiple comparisons (Two-stage step-up method of Benjamini, Krieger and Yekutieli; Q = 0.05; 6 comparisons with WT condition; q value is shown). Mean and S.D. error bars. n = 3 independent biological replicates.

D - Additional compound KO clones from Figure 7D.

Figure S5

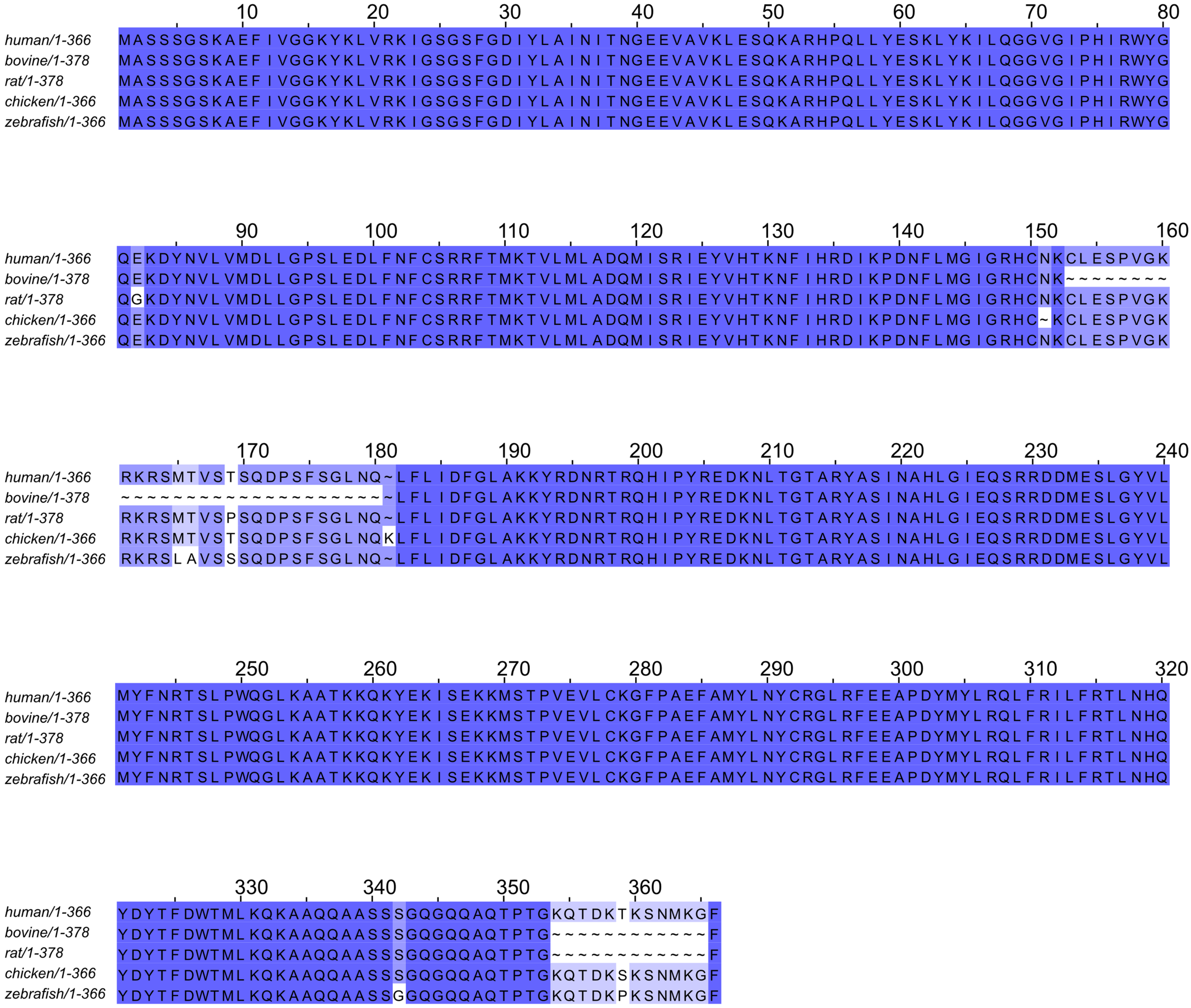

Figure S5

Protein sequence alignment of selected CK1α splice variants of species from Table 2. Alignment was created in Jalview (94). Color code reflects conservation. Human CK1α is CK1α-v1.

SUPPORTING INFORMATION - uncropped WB membranes of Figure 3B

Copy of **Figure 3B** for convenient comparison.

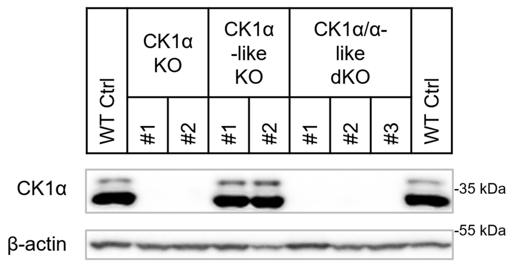

signal (10s exposition)

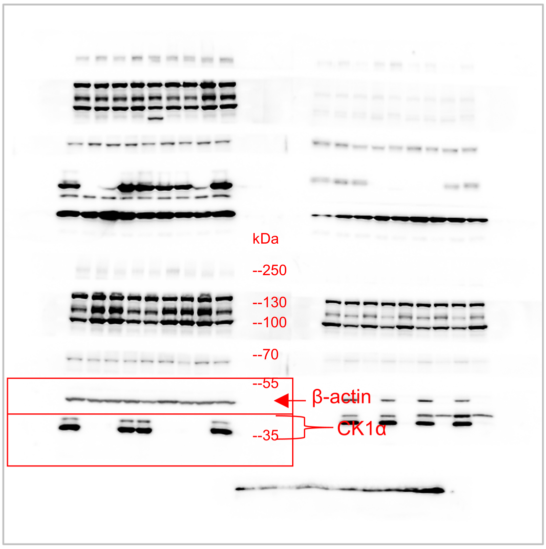

marker + signal (1m exposition)

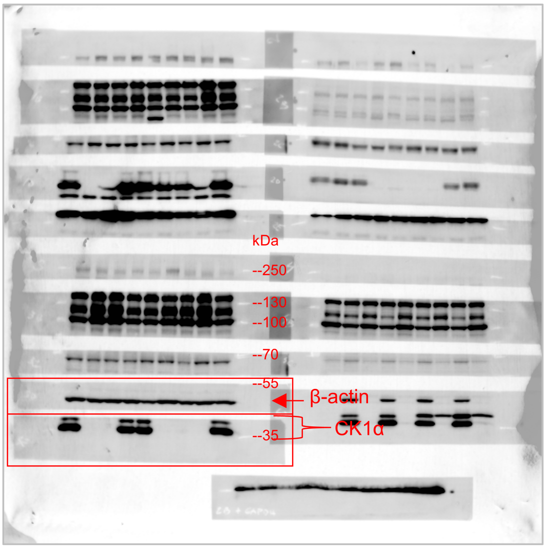

SUPPORTING INFORMATION - uncropped WB membranes of Figure 3D & 7A

Copy of **Figure 3D** for convenient comparison.

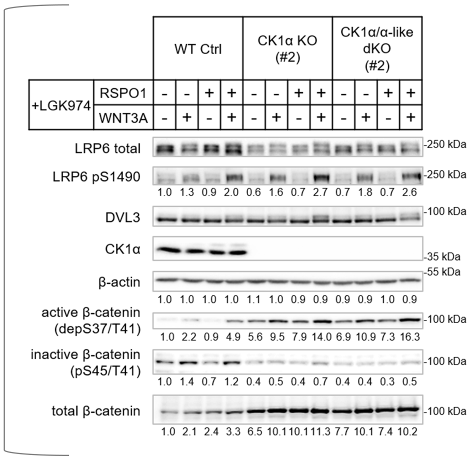

Copy of **Figure 7A** for convenient comparison.

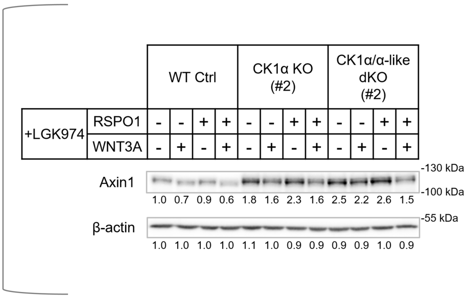

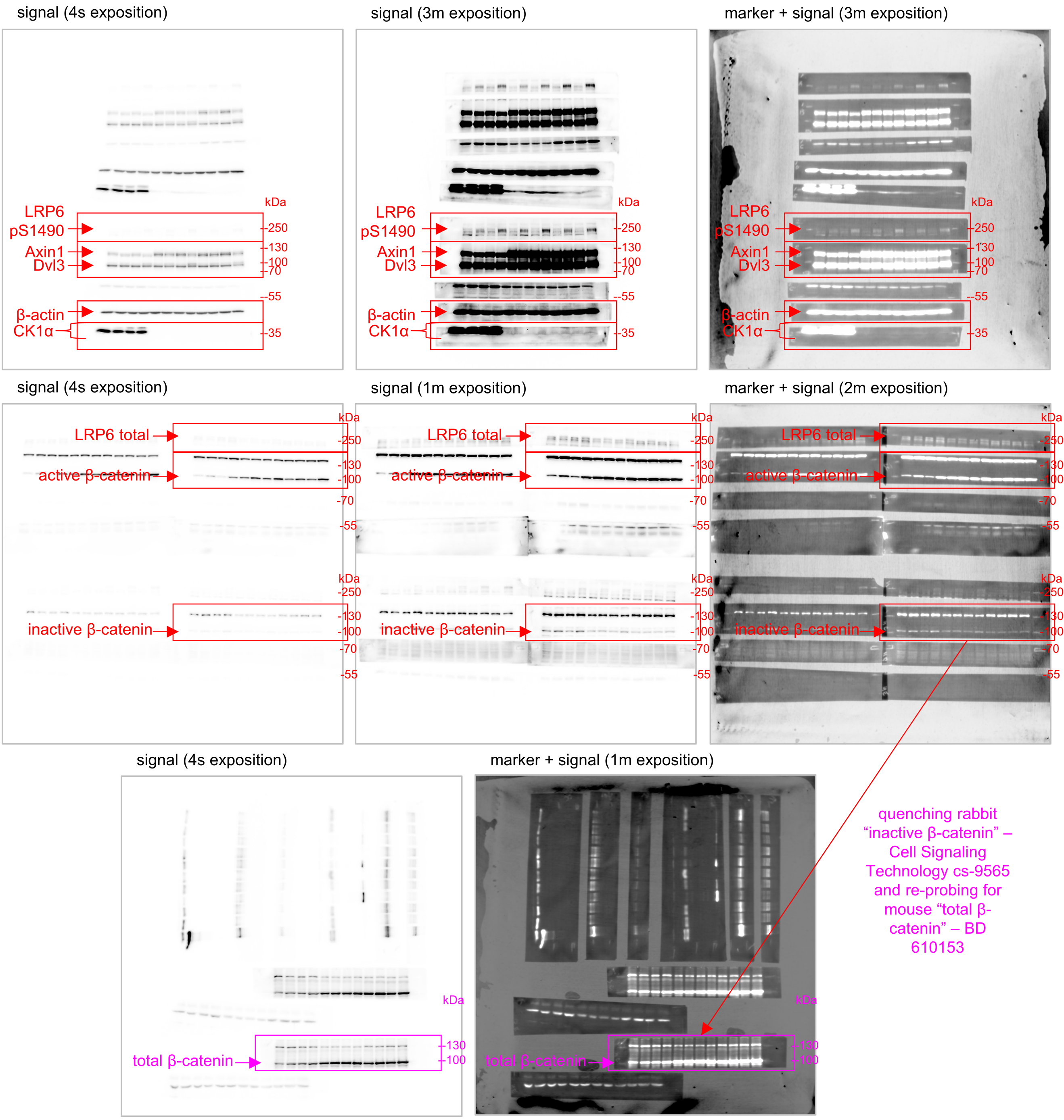

SUPPORTING INFORMATION - uncropped WB membranes of Figure 6C

Copy of **Figure 6C** for convenient comparison.

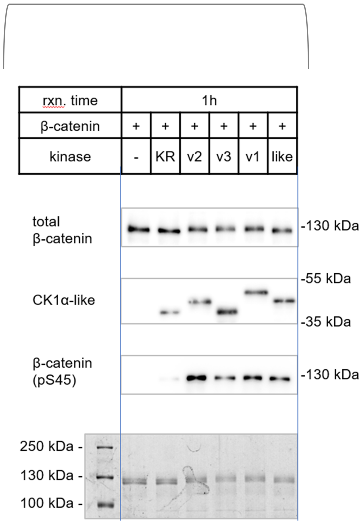

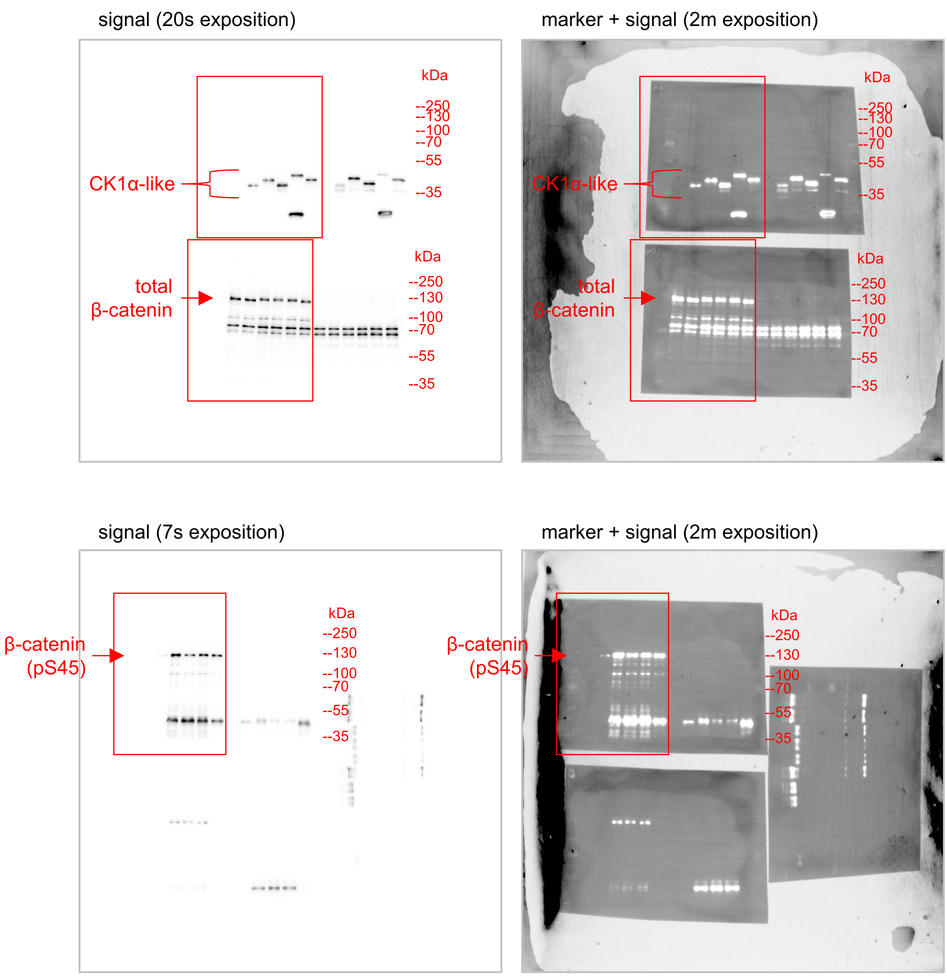

Figure 6C - whole photo of stained SDS gel

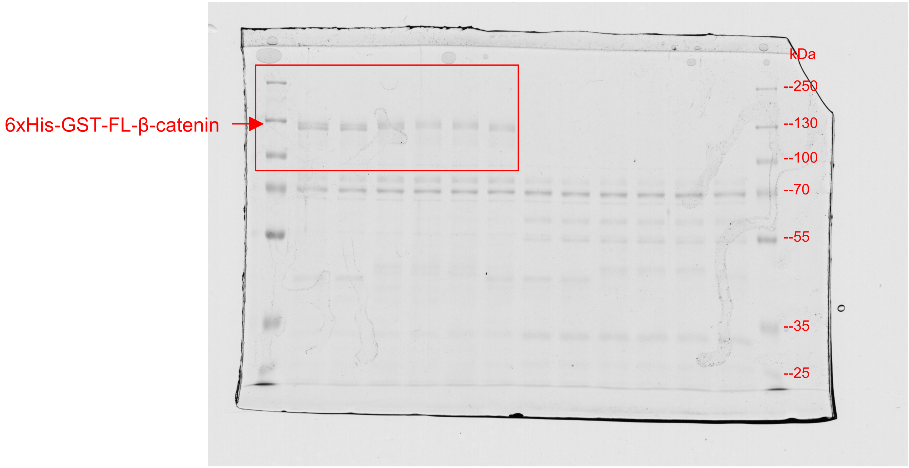

SUPPORTING INFORMATION - uncropped WB membranes of Figure 7D (left panel)

Copy of **Figure 7D** for convenient comparison. Uncropped membranes for the left panel are shown underneath.

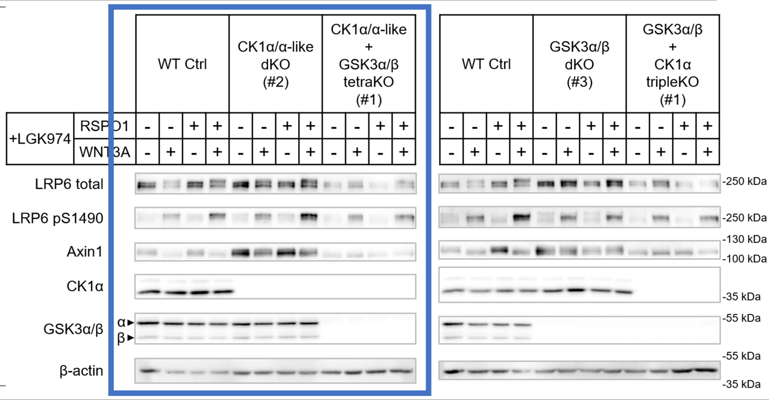

signal (45s exposition)

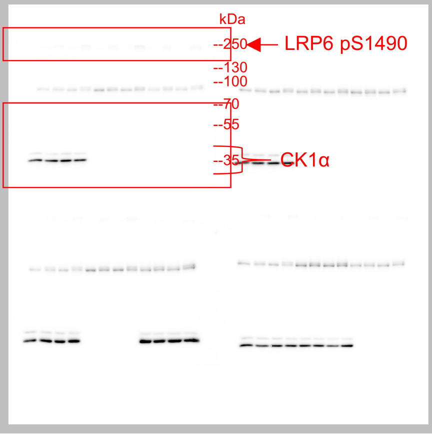

signal (7m exposition)

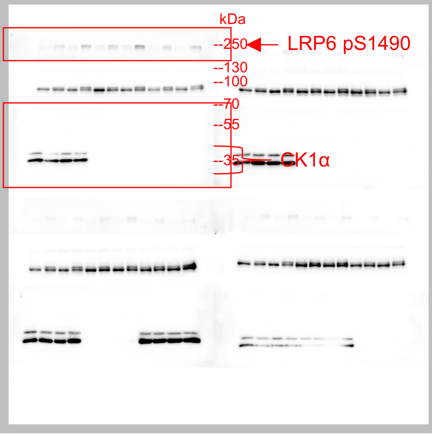

marker + signal (7m exposition)

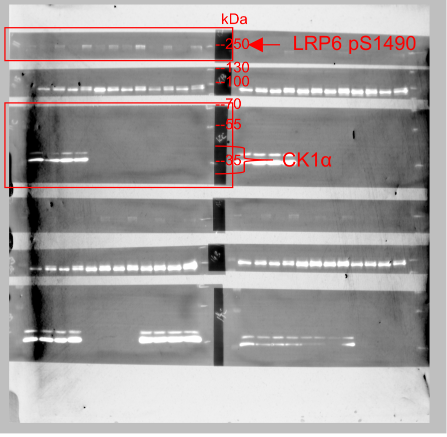

signal (10m exposition) RE-PROBING

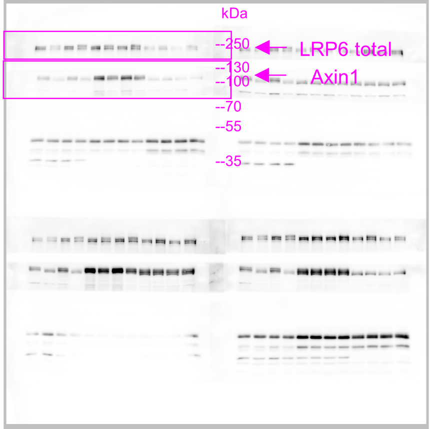

signal (40s exposition) RE-PROBING

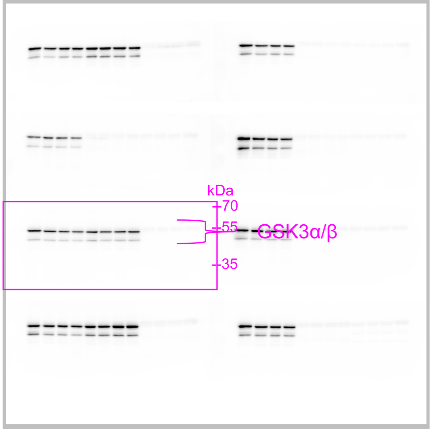

signal (2m exposition) RE-PROBING

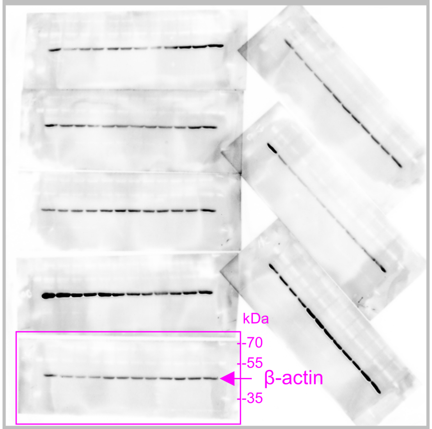

marker + signal (10m exposition)

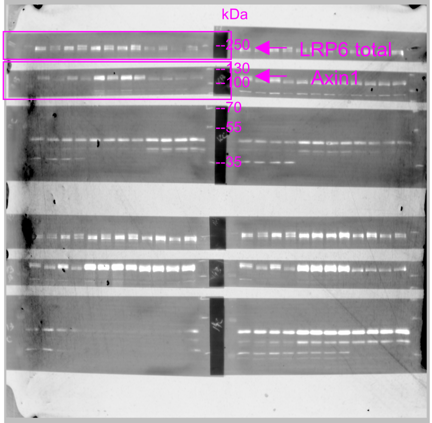

marker + signal (3m exposition)

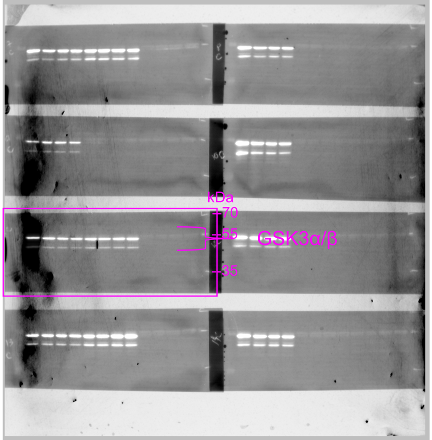

marker + signal (4m exposition)

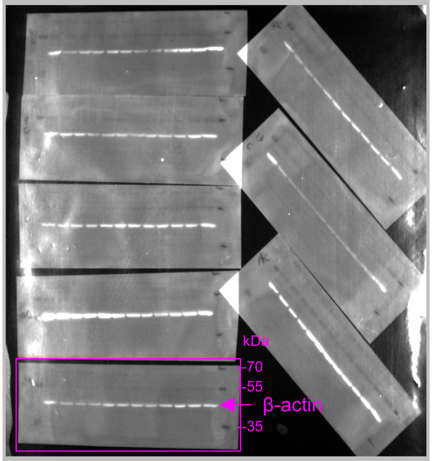

SUPPORTING INFORMATION - uncropped WB membranes of Figure 7D (right panel)

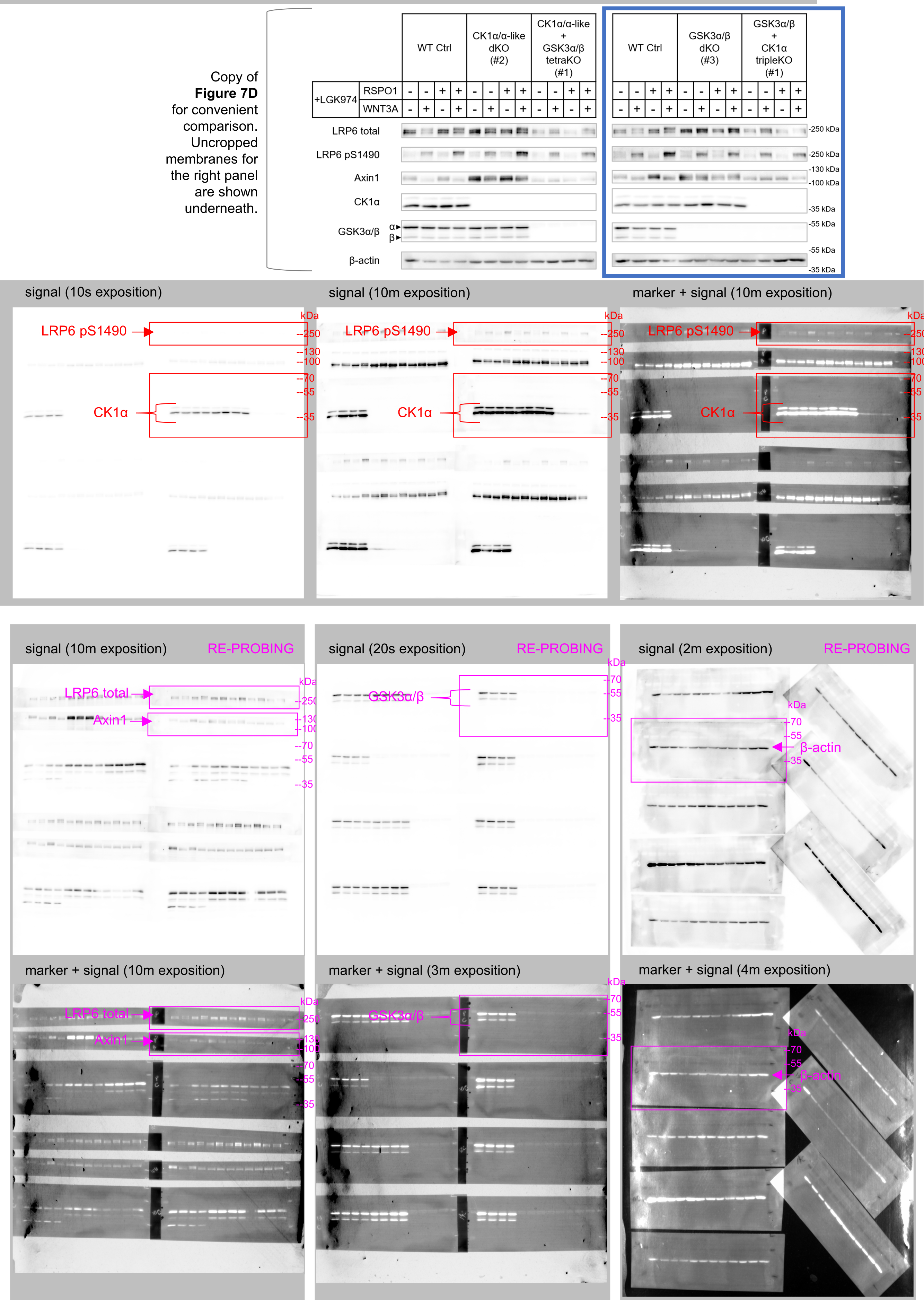

SUPPORTING INFORMATION - uncropped WB membranes of Figure S3B (upper panel)

Copy of **Figure S3B (upper panel)** for convenient comparison.

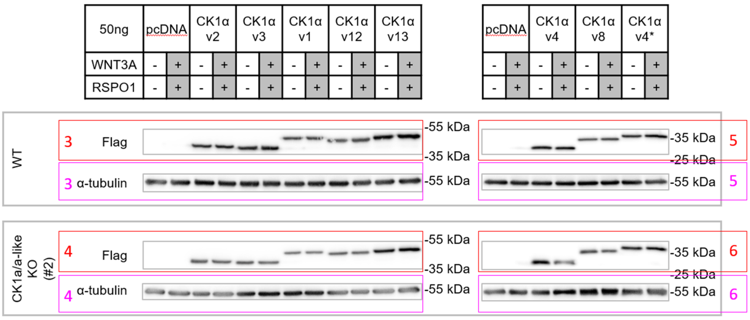

signal (10s exposition)

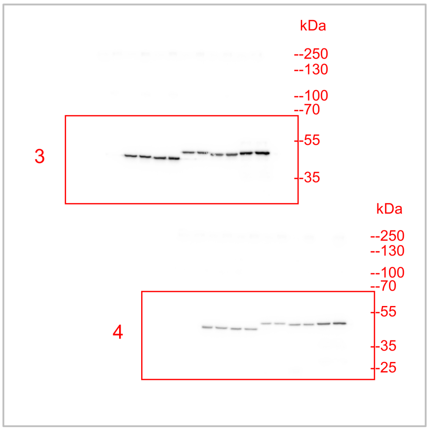

marker + signal (10m exposition)

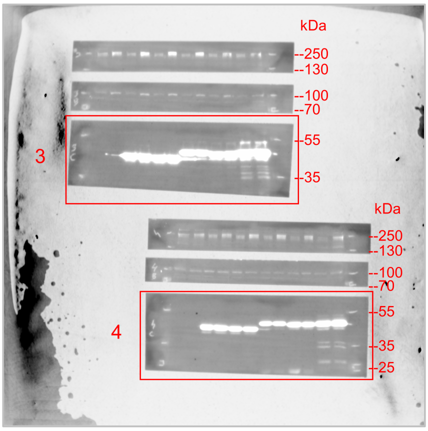

signal (10s exposition)

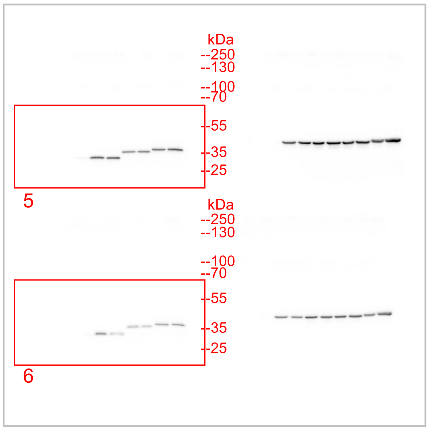

marker + signal (10m exposition)

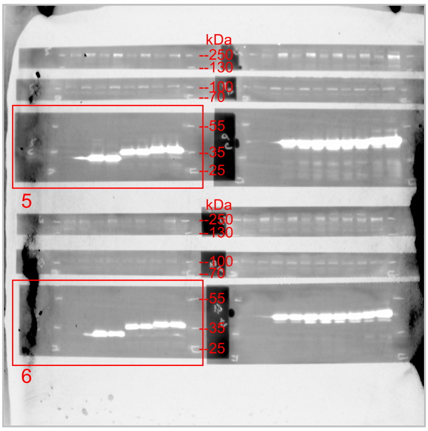

signal (10s exposition)

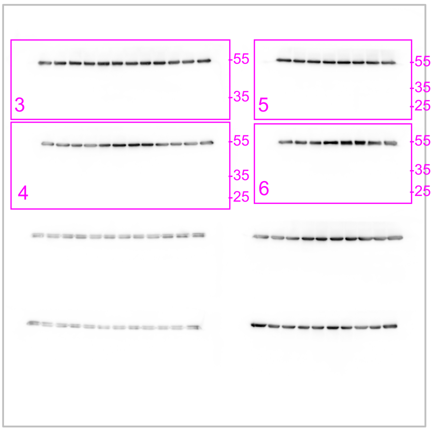

marker + signal (10m exposition)

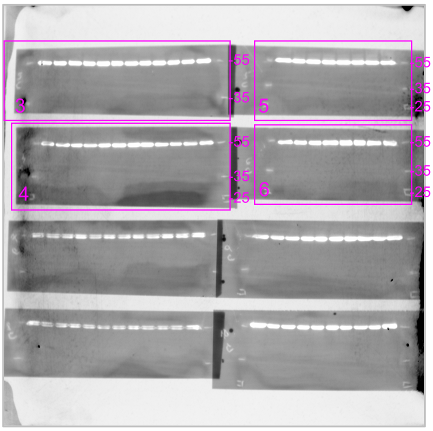

RE-PROBING

SUPPORTING INFORMATION - uncropped WB membranes of Figure S3B (lower panel)

Copy of **Figure S3B (lower panel)** for convenient comparison.

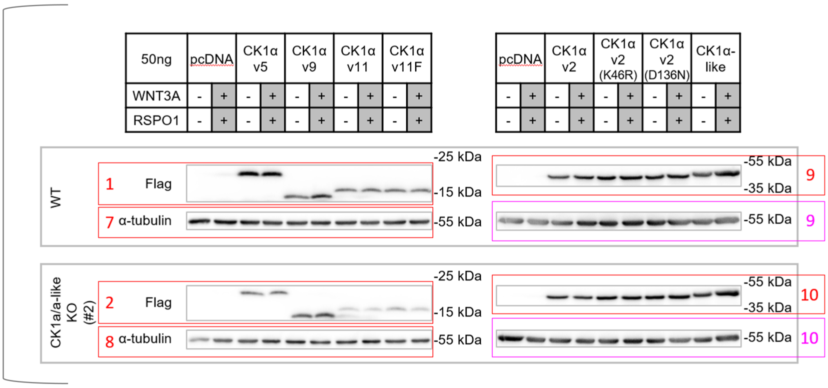

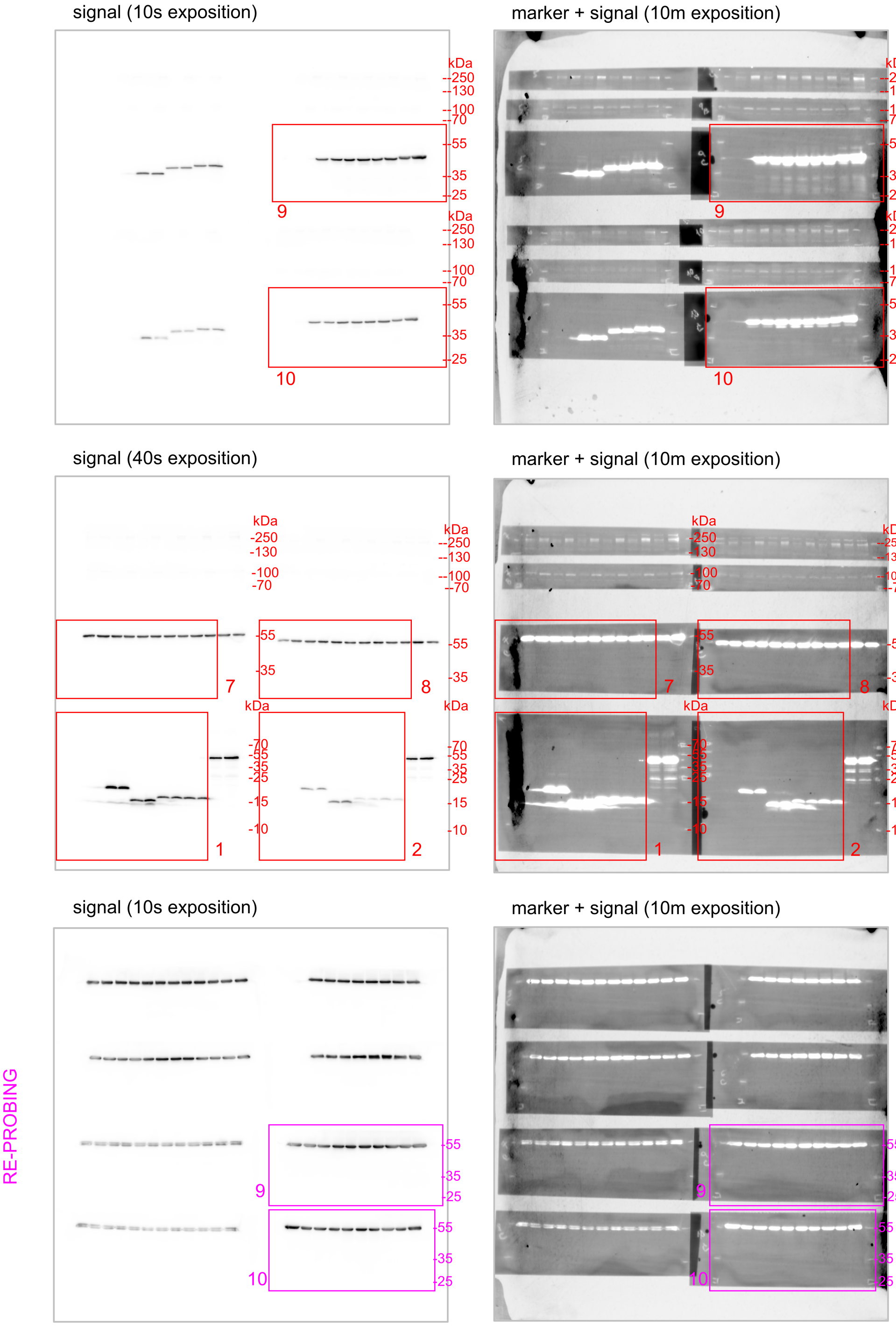

SUPPORTING INFORMATION - uncropped WB membranes of Figure S4D (left panel)

Copy of **Figure S4D** for convenient comparison. Uncropped membranes for the left panel are shown underneath.

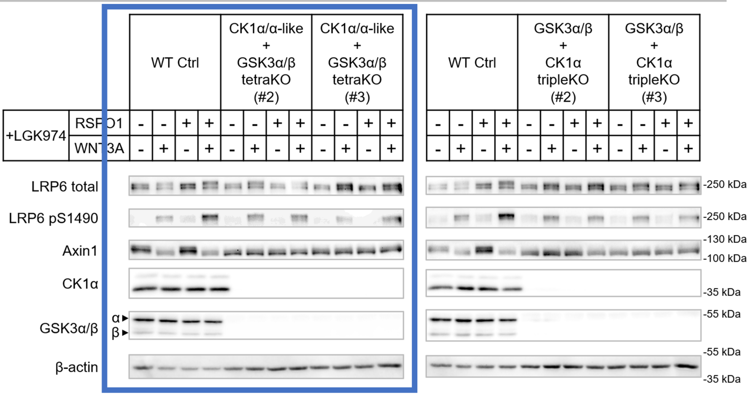

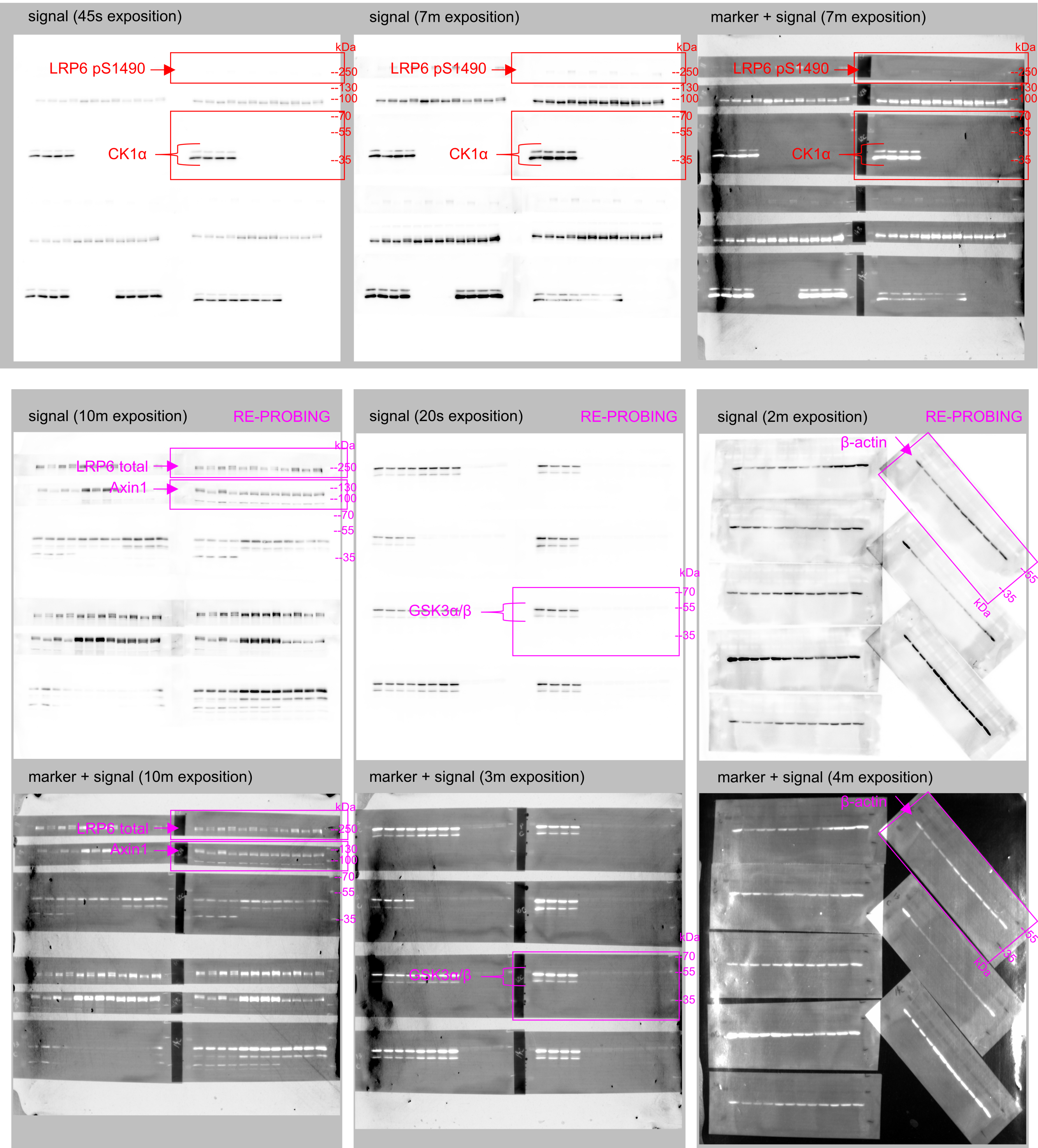

SUPPORTING INFORMATION - uncropped WB membranes of Figure S4D (right panel)

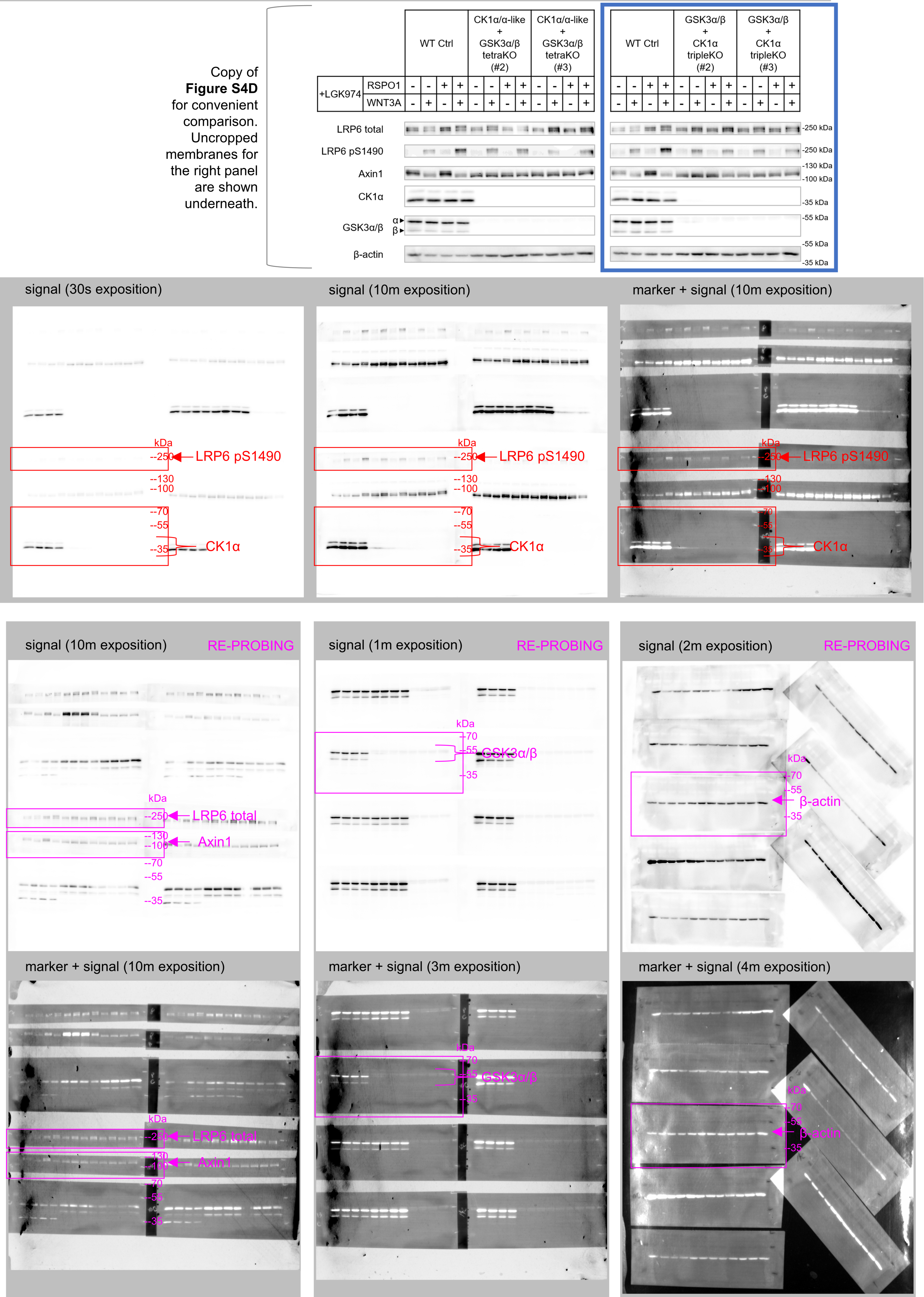

Supplement: Supplemental Figures S1–S5 [file mmc1.pdf]
